# Epidemiological evidence for associations between variants in CHRNA genes and risk of lung cancer and chronic obstructive pulmonary disease

**DOI:** 10.3389/fonc.2022.1001864

**Published:** 2022-10-06

**Authors:** Lei Yang, Zelin Yang, Chunjian Zuo, Xiaolong Lv, Tianyu Liu, Chenhao Jia, Huanwen Chen

**Affiliations:** ^1^ Department of Cardiothoracic Surgery, The First Affiliated Hospital of Chongqing Medical University, Chongqing, China; ^2^ Department of Thoracic Surgery, Army Medical Center of People’s Liberation Army of China (PLA), Chongqing, China

**Keywords:** CHRNA, genetic variants, lung cancer, COPD, susceptibility

## Abstract

**Background:**

Genetic studies have previously reported that single-nucleotide polymorphisms (SNPs) in *CHRNA* genes (such as *CHRNA3*, *CHRNA4*, *CHRNA5*, or *CHRNA3-CHRNA5-CHRNB4* clusters) are linked to the risk of neoplastic and non-neoplastic diseases. However, these conclusions were controversial and no systematic research synopsis has been available. We aimed to synthesize current knowledge of variants in the *CHRNA* genes on the risk of diseases.

**Methods:**

We systematically searched for publications using PubMed, Medline, and Web of Science on or before 25 August 2021. A total of 1,818 publications were identified, of which 29 were deemed eligible for inclusion that could be used to perform meta-analysis based on at least three data sources to assess whether the morbidity associated with neoplastic and non-neoplastic diseases can be attributed to SNPs in *CHRNA* genes. To further evaluate the authenticity of cumulative evidence proving significant associations, the present study covered the Venice criteria and false-positive report probability tests. Through the Encyclopedia of DNA Elements (ENCODE) project, we created functional annotations for strong associations.

**Results:**

Meta-analyses were done for nine genetic variants with two diseases {chronic obstructive pulmonary disease (COPD) and lung cancer (LC)}that had at least three data sources. Interestingly, eight polymorphisms were significantly related to changes in the susceptibility COPD and LC (*p* < 0.05). Of these, strong evidence was assigned to six variants (28 significant associations): *CHRNA3* rs1051730, *CHRNA3* rs6495309, and *CHRNA5* rs16969968 with COPD risk, and *CHRNA3* rs1051730, *CHRNA3* rs578776, *CHRNA3* rs6495309, *CHRNA3* rs938682, *CHRNA5* rs16969968, and *CHRNA5* rs588765 with LC risk; moderate evidence was assigned to five SNPs (12 total associations) with LC or COPD risk. Data from ENCODE and other public databases showed that SNPs with strong evidence may be located in presumptive functional regions.

**Conclusions:**

Our study summarized comprehensive evidence showing that common mutations in *CHRNA* genes are strongly related to LC and COPD risk. The study also elucidated the vital function of *CHRNA* genes in genetic predispositions to human diseases.

## 1. Introduction

Worldwide, many diseases, including neoplastic and non-neoplastic illnesses, have become significant obstacles to the progress of human society. Although lung cancer (LC) has been surpassed by female breast cancer and is the second most commonly diagnosed cancer, the maximum number of deaths in cancerous people is attributed to LC (1). According to a report in 2020, the mortality rate of LC is 18%, with an estimated 1.8 million deaths per year, which exceeds other cancers by far (1). Chronic obstructive pulmonary disease (COPD) is another common global disease. The disease is considered preventable and remediable, and it is characterized by irreversible airway obstruction (2). According to various studies, environmental factors (such as tobacco smoking, ionizing radiation, occupational exposures, and air pollution for LC, and tobacco smoke, occupational dust, vapours, and fumes air pollutants for COPD) and variations in genes facilitate the advancement of LC and COPD (3, 4). Furthermore, more than 80% of LC patients have smoked, and about 50% of COPD cases are related to tobacco smoke worldwide (5–7). However, not everyone exposed to these risk factors develops LC, COPD, or other diseases, and only 20% of smokers are confirmed to have LC or COPD. In fact, previous studies have suggested that genetic variants may be responsible for susceptibility to LC and COPD (8, 9).

According to existing studies, the *CHRNA* gene can encode nicotine receptors that are expressed in many cells. These receptors can combine with their ligands (e.g., acetylcholine) to transmit biological information. Nicotine is an alkaloid found in tobacco that mimics acetylcholine (10). According to published articles, there are abundant nicotine receptors, which are thought to be the reason for nicotine addiction, in the brain core (11). In addition, because nicotine receptors consist of lung epithelial lung cells, tobacco carcinogens are presumed to act as risk factors for the onset of LC, and the receptors’ signal transduction pathway may facilitate tumor metastasis (12–14). In previous papers, *CHRNA3* and *CHRNA5* have been proven to have significant correlations with smokers’ susceptibility to LC for their polymorphisms (15). The hypomethylation in the promoter region of *CHRNB4* on 15q25 resulted in tumors’ transcript overexpressing, and there were significant hypermethylation expression changes in *CHRNA3* and telomerase reverse transcriptase (*TERT*) and potential tumor suppressor genes that played out due to frequent methylation events (16, 17). Genome-wide association studies (GWAS) have also demonstrated that single-nucleotide polymorphisms (SNPs, such as CHRNA3, CHRNA5, and IREB2) in an area of chromosome 15q25 are closely related to COPD (18, 19). Cigarette smoking, the primary risk factor for the development of COPD, causes the chronic inflammatory process that promotes the structural changes in the small airways and parenchyma (20). The exchange of *CHRNA5* transcript expression may have influenced the inflammatory response to smoking (21).

In 2003, Chou et al. reported that neuronal nicotinic acetylcholine receptor subunit alpha 4 (*CHRNA4*) polymorphisms can play a role in febrile convulsions (22). In 2008, Amos et al. performed a GWAS in Caucasians and found that rs1051730 in *CHRNA3* was significantly related to LC susceptibility (23). In 2009, a study by Falvella et al. determined that both *CHRNA3* (a slight downgrade) and *CHRNA5* (a significant increase) expressed differently in lung adenocarcinoma tissue, which further explains the role of *CHRNA* SNPs in LC onset (24). Since then, studies have revealed the relationship between LC susceptibility and *CHRNA* SNPs, including rs12914385, rs3743073, rs578776, rs6495309, rs8042374, and rs938682 in *CHRNA3* and rs16969968 and rs588765 in *CHRNA5*. In 2012, Yang et al. declared that the rs6495309 CC genotype and rs6495309 CT/CC variant genotypes could increase the morbidity of LC and COPD in China (25). In the same year, Lee et al. reported that the CT or TT genotypes of rs6495309 in *CHRNA3* could significantly decrease the risk of COPD in the Korean population (26).

Although several studies have reported significant associations between *CHRNA* SNPs and the risk of cancerous or non-cancerous diseases, some studies have held controversial or disputed opinions about the same *CHRNA* SNPs. The possible reasons may have included small sample sizes or inauthentic positive associations. Because a comprehensive research synopsis with systematic functional annotation has yet to measure the epidemiological evidence of genetic associations between *CHRNA* genes and susceptibility to cancerous or non-cancerous diseases, the present study aimed to account for the effect of studied *CHRNA* SNPs on the risk of all types of cancerous or non-cancerous diseases. First, we conducted a meta-analysis using data collected from all relevant existing studies. We then detected the statistical power of the generated significant evidence. Finally, we conducted a systematic functional annotation to detect the molecular mechanisms of the approved connections.

## 2. Methods

This study was performed under the guidance of the Preferred Reporting Items for Systematic Reviews and Meta-Analyses Statement guidelines (**Supplementary Table S1**) and the Human Genome Epidemiology Network for the systematic review of genetic association studies (27, 28).

### 2.1 Literature search

We used PubMed, Web of Science, and Medline to search for relevant papers delivered before 25 Aug 2021 by employing the following terms: *CHRNA3* or *CHRNA*4 or *CHRNA5* or *CHRNA3*-*CHRNA5*-*CHRNB4* cluster and variant or variation or polymorphism or genotype or single-nucleotide polymorphism or SNP or mutation or rs. The published years ranged from 2008 to 2020. In addition, the references of the qualified articles were checked to acquire other related data.

### 2.2 Criteria for inclusion and exclusion

We selected qualified studies that met the following criteria: (i) studies that discussed relationships between *CHRNA* SNPs with the risk of neoplastic or non-cancerous disease with case–control or cohort designed studies of humanity; (ii) studies that provided the sample sizes of cases and controls, respectively; when necessary, studies could provide the amount of genotype and/or allelic distributions to compute the values of odds ratios (ORs) and 95% confidence intervals (95% CIs); and (iii) the full text of the journals was written completely in English. Any article that satisfied any of the following criteria were excluded: (i) studies without sufficient relevant data; (ii) studies in the form of conference abstracts, meetings, or letters to editors; and (iii) studies focused only on the prognoses and survival of cancer patients rather than on cancer morbidity.

### 2.3 Data extraction

We assigned two different authors to collect correlative information and cross-check each other. Any nonconformity was discussed with the corresponding author and eventually resolved. The following details would be recorded when the qualified SNPs were found: publication year, first author, SNP number, ethnicity, study design, gene name, gene variation, sample sizes of cases and controls, genotype counts, and minor allelic frequency. Ethnicity comprised four categories: Asians (East Asian descent), Caucasians (European descent), Africans (African descent), or others (including people from other countries, such as Indians, Native Hawaiians, Latinos, Hispanics, or mixed). More than 80% of the study’s subjects belonged to one of the above-mentioned groups, and the overall population was composed of two or more of these groups. In addition, we selected the study that had been published most recently, which had the most complete sample of participants and the greatest amount of at least two studies that included the same study population. Because the presentation forms of results were usually inconsonant when identical genetic variants were studied in different research, we collated it at the NCBI site (https://www.ncbi.nlm.nih.gov/snp/) and then chose the most up-to-date and consistent one.

### 2.4 Statistical analysis

This study employed three models—the allelic model, the dominant model, and the recessive model (**Supplementary Table S2**)—to put the comprehensive meta-analyses into effect. An ethnicity-based subgroup analysis was also implemented as needed. The heterogeneity among the different publications was evaluated with the *I^2^* statistic and Cochran’s *Q* test (29, 30). Three different ranges of *I*²values were given different means: ≤25% (without or little heterogeneity), 25%–50% (middling heterogeneity), and ≥50% (abundant heterogeneity). A different kind of model was employed according to the *p*-value generated from the *Q* statistic. The random effect model was adopted if the *p*-value was < 0.1, and the fixed effect model was used if the *p*-value was > 0.1. In order to further evaluate the reliability of the significant ORs, we worked out sensitivity analyses for all SNPs with significant associations by excluding a single study (or dataset), the controls of studies that did not match the Hardy–Weinberg equilibrium (HWE), and the first published study. The study evaluated potential publication bias and small-study bias according to Begg’s test and Egger’s test, respectively (31, 32). The study also calculated the chance of collecting too many statistical findings for an independent meta-analysis (with a significance level of *p* < 0.1) (33). The small-study bias and potential publication bias were evaluated by adopting Begg’s test and a modified version of Egger’s test (with a significance level of *p* < 0.1, as recommended) (30, 31). Stata version 12 was used to conduct the statistical analyses (Stata, College Station, TX, USA).

### 2.5 Evaluation of cumulative evidence

To assess the epidemiological credibility of nominally statistical associations proved by meta-analyses, the present study used the Venice criteria to rate the cumulative evidence *via* three levels (strong, moderate, or weak) (34). The criteria consist of the amount of evidence, protection from bias, and replication of association (graded as A, B, or C, respectively). First, there were three levels for the quantity of evidence based on the total quantity of alleles or genotypes among the cases and controls. These levels were distinguished as follows: >1,000 (level A), 100–1,000 (level B), and <100 (level C). Similarly, the replication of association was evaluated by heterogeneity statistics (*I* ^2^) and was rated according to the following three levels: *I* ^2^ ≤ 25% (level A), 25% < *I* ^2^ < 50% (level B), and *I* ^2^ ≥ 50% (level C). Finally, we used sensitivity analysis, publication bias, the chance of collecting too many statistical findings, and small-study bias to assess the protection from bias. Importantly, associations without observable biases were assigned to grade A, and a grade A criterion meant that making an association clear was improbable. Grade B was chosen if an association lacked crucial information on identifying evidence without the presence of distinct bias (35). Finally, an association was assigned to grade C if the bias was explicit or the act of making the association clear was improbable.

The Venice criteria also cover an abundant checklist for checking the sources of bias in different options (see supplementary information notes). In addition, the confidence level of an association is related to the evaluation of protection from bias. To illustrate, a summary OR of an association of <1.15 (OR > 0.87 in a protection effect) was categorized as level C unless the relationship had been proven in other studies without obvious publication bias (primarily, GWAS or GWAS meta-analyses from collaborative studies). Finally, cumulative epidemiological evidence of nominally statistical associations was divided into three groups: strong associations (all three grades were A), weak associations (at least one grade of C), and moderate associations (apart from the above).

As Wacholder et al. recommended, a prior probability of 0.05 and a false-positive report probability (FPRP) cutoff value of 0.2 in the FPRP assay must be calculated to find potentially false-positive results among statistical associations and to discuss whether they are supposed to be excluded (36). We used the Excel spreadsheet acquired from the website to calculate FPRP values (35). We considered an association notable when the FPRP value was below the prespecified noteworthiness value of 0.2, indicating that the association might be true. The true evidence was graded by FPRP values of <0.05, 0.05–0.2, and >0.2, indicating strong, moderate, and weak, respectively. With a strong magnitude of FPRP, the credibility of the evidence would be upgraded from weak to moderate or from moderate to strong. If the FPRP was assigned as weak, we would downgrade the credibility of association.

### 2.6 Functional annotation

The underlying functional role of variants in *CHRNA* genes was evaluated with information from the Encyclopedia of DNA Elements (ENCODE) tool, HaploReg v. 4.1, and the UCSC Genome browser (http://genome.ucsc.edu/) (37). Furthermore, the present work explored genome-wide cis-eQTL data in multiple tissues from the Genotype-Tissue Expression Project and the Multiple Tissue Human Expression Resource Project databases to reveal whether these genes could account for the observed findings in these loci (38, 39).

## 3. Results

### 3.1 Characteristics of eligible studies

**Figure 1:**
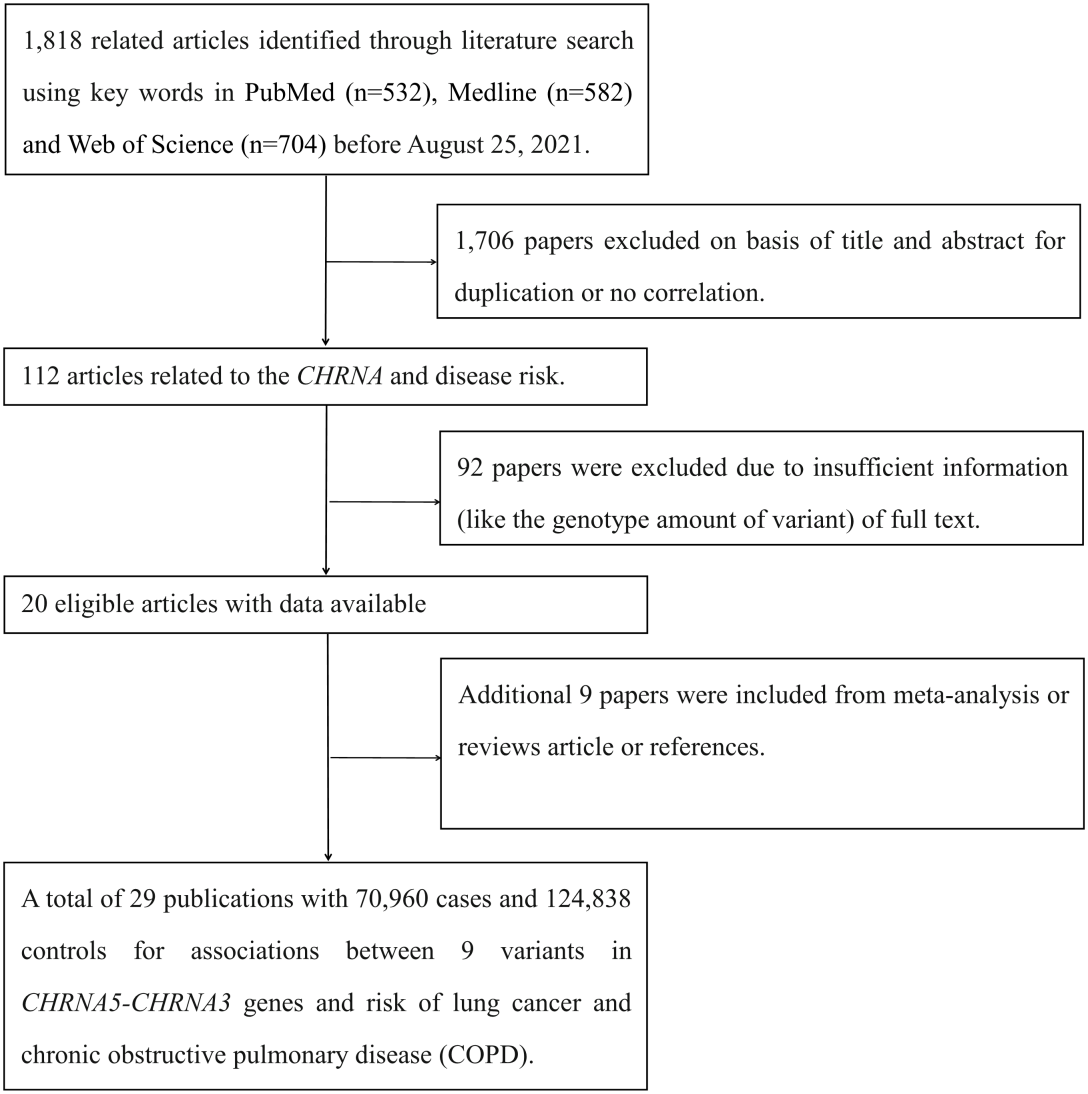
Flow diagram of literature search and study selection.

We initially searched for 1,818 studies using PubMed, Medline, and Web of Science (**Figure 1**). Of these, 1,706 papers were excluded because the titles and abstracts were duplicates or lacked correlation, and 92 papers were excluded due to insufficient information (such as about the number of variants in a genotype) in the full text. In addition, nine papers were included from meta-analyses, review articles, or references. The present study included a total of 29 publications with 70,960 cases and 124,838 controls to evaluate the relationship between nine *CHRNA* SNPs and LC or COPD susceptibility (eight SNPs with a relationship to LC and three SNPs with a relationship to COPD, respectively) after filtering out SNPs with no more than two datasets. The demographic characteristics of all available publications are summarized in **Supplementary Table S3**. Multiple diseases (such as epilepsy, Parkinson’s disease, and Alzheimer’s disease) were not evaluated by meta-analysis because there was only one dataset for each disease.

### 3.2 Main meta-analyses

Meta-analyses were performed to evaluate the associations between nine *CHRNA* SNPs and the risk of COPD or LC (**Table 1**). Of these, eight SNPs were statistically associated with the risk of LC or COPD risk; three SNPs were nominally statistically related to COPD risk (rs1051730 and rs6495309 in *CHRNA3* and rs16969968 in *CHRNA5*); and eight SNPs were nominally statistically associated with LC risk (rs1051730, rs12914385, rs578776, rs6495309, rs8042374, and rs938682 in *CHRNA3* and rs16969968 and rs588765 in *CHRNA5*).

**Table 1:** Genetic variants showing summary ORs for different disease risks in main meta-analyses in all three genetic models.

Gene	Variant	Alleles^a^	Ethnicity	Number evaluated		Genetic model	Effect model	Risk of meta-analysis		Heterogeneity		Venice criteria^b^	FPRP values^c^	Credibility of evidence^d^
				Datasets	Cases/Controls			OR (95% CI)	*p*-value	*I* ^2^ (%)	*P_Q_*			
**Chronic obstructive pulmonary disease (COPD)**														
CHRNA3	rs1051730	A vs. G	Overall	4	1,618/1,909	Allelic	Fixed	1.630 (1.293–2.054)	<0.001	0.0	0.897	BAA	<0.001	Strong
CHRNA3	rs1051730	A vs. G	Overall	4	1,618/1,909	Dominant	Fixed	1.662 (1.300–2.124)	<0.001	0.0	0.877	BAA	<0.001	Strong
CHRNA3	rs1051730	A vs. G	Overall	4	1,618/1,909	Recessive	Random	2.433 (0.737–8.033)	0.144	0.0	0.425			
CHRNA3	rs1051730	A vs. G	Asian	3	1,501/1,717	Allelic	Fixed	1.591 (1.204–2.103)	0.001	0.0	0.780	BAA	0.001	Strong
CHRNA3	rs1051730	A vs. G	Asian	3	1,501/1,717	Dominant	Fixed	1.625 (1.222–2.160)	0.001	0.0	0.748	BAA	<0.001	Strong
CHRNA3	rs1051730	A vs. G	Asian	3	1,501/1,717	Recessive	Random	1.017 (0.106–9.793)	0.988	0.0	0.341			
CHRNA3	rs6495309	T vs. C	Asian	3	1,917/2,068	Allelic	Fixed	0.830 (0.759–0.906)	<0.001	25.9	0.259	ABA	<0.001	Strong
CHRNA3	rs6495309	T vs. C	Asian	3	1,917/2,068	Dominant	Fixed	0.736 (0.644–0.842)	<0.001	0.0	0.892	AAA	<0.001	Strong
CHRNA3	rs6495309	T vs. C	Asian	3	1,917/2,068	Recessive	Random	0.830 (0.637–1.081)	0.166	64.7	0.059			
CHRNA5	rs16969968	A vs. G	Overall	6	3,126/7,685	Allelic	Fixed	1.307 (1.205–1.417)	<0.001	0.0	0.471	AAC	<0.001	Moderate
CHRNA5	rs16969968	A vs. G	Overall	6	3,126/7,685	Dominant	Fixed	1.413 (1.268–1.573)	<0.001	0.0	0.687	AAC	<0.001	Moderate
CHRNA5	rs16969968	A vs. G	Overall	6	3,126/7,685	Recessive	Fixed	1.370 (1.154–1.625)	<0.001	0.0	0.713	BAA	<0.001	Strong
CHRNA5	rs16969968	A vs. G	Asian	3	1,501/1,717	Allelic	Fixed	1.591 (1.204–2.103)	0.001	0.0	0.780	BAA	0.001	Strong
CHRNA5	rs16969968	A vs. G	Asian	3	1,501/1,717	Dominant	Fixed	1.625 (1.222–2.160)	0.001	0.0	0.748	BAA	<0.001	Strong
CHRNA5	rs16969968	A vs. G	Asian	3	1,501/1,717	Recessive	Fixed	1.017 (0.143–7.229)	0.987	0.0	0.341			
**Lung cancer**														
CHRNA3	rs1051730	A vs. G	Overall	11	7,657/7,515	Allelic	Fixed	1.348 (1.276–1.424)	<0.001	37.0	0.103	ABA	<0.001	Strong
CHRNA3	rs1051730	A vs. G	Overall	11	7,657/7,515	Dominant	Fixed	1.446 (1.342–1.559)	<0.001	10.0	0.349	AAA	<0.001	Strong
CHRNA3	rs1051730	A vs. G	Overall	11	7,657/7,515	Recessive	Fixed	1.519 (1.356–1.700)	<0.001	0.0	0.582	AAA	<0.001	Strong
CHRNA3	rs1051730	A vs. G	Asian	3	1,834/1,460	Allelic	Fixed	2.280 (1.626–3.197)	<0.001	0.0	0.978	BAA	<0.001	Strong
CHRNA3	rs1051730	A vs. G	Asian	3	1,834/1,460	Dominant	Fixed	2.329 (1.649–3.291)	<0.001	0.0	0.982	BAA	<0.001	Strong
CHRNA3	rs1051730	A vs. G	Asian	3	1,834/1,460	Recessive	Random	2.249 (0.234–21.659)	0.483	Na	Na			
CHRNA3	rs1051730	A vs. G	Caucasian	6	3,492/5,434	Allelic	Fixed	1.313 (1.240–1.390)	<0.001	0.0	0.611	AAA	<0.001	Strong
CHRNA3	rs1051730	A vs. G	Caucasian	6	3,492/5,434	Dominant	Fixed	1.389 (1.283–1.504)	<0.001	0.0	0.886	AAA	<0.001	Strong
CHRNA3	rs1051730	A vs. G	Caucasian	6	3,492/5,434	Recessive	Fixed	1.506 (1.344–1.688)	<0.001	8.0	0.365	AAA	<0.001	Strong
CHRNA3	rs12914385	T vs. C	Overall	4	10,037/4,443	Allelic	Random	1.096 (0.799–1.505)	0.569	96.7	< 0.001			
CHRNA3	rs12914385	T vs. C	Overall	4	10,037/4,443	Dominant	Random	1.101 (0.712–1.703)	0.665	96.6	< 0.001			
CHRNA3	rs12914385	T vs. C	Overall	4	10,037/4,443	Recessive	Random	1.206 (0.839–1.733)	0.312	89.4	< 0.001			
CHRNA3	rs12914385	T vs. C	Caucasian	3	8,514/2,900	Allelic	Random	1.264 (1.053–1.517)	0.012	84.4	0.002	ACA	0.012	Moderate
CHRNA3	rs12914385	T vs. C	Caucasian	3	8,514/2,900	Dominant	Random	1.458 (1.331–1.596)	0.014	81.5	0.004	ACA	<0.001	Moderate
CHRNA3	rs12914385	T vs. C	Caucasian	3	8,514/2,900	Recessive	Random	1.449 (1.134–1.851)	0.003	66.5	0.051	ACA	0.003	Moderate
CHRNA3	rs3743073	T vs. G	Overall	3	1,391/1,500	Allelic	Random	0.785 (0.546–1.129)	0.191	91.3	< 0.001			
CHRNA3	rs3743073	T vs. G	Overall	3	1,391/1,500	Dominant	Random	0.755 (0.410–1.392)	0.368	90.7	< 0.001			
CHRNA3	rs3743073	T vs. G	Overall	3	1,391/1,500	Recessive	Random	0.729 (0.504–1.056)	0.094	80.0	0.007			
CHRNA3	rs578776	A vs. G	Overall	3	1,254/2,009	Allelic	Fixed	0.868 (0.773–0.976)	0.018	0.0	0.908	AAA	0.018	Strong
CHRNA3	rs578776	A vs. G	Overall	3	1,254/2,009	Dominant	Fixed	0.841 (0.722–0.979)	0.026	0.0	0.937	AAA	0.025	Strong
CHRNA3	rs578776	A vs. G	Overall	3	1,254/2,009	Recessive	Random	0.839 (0.655–1.073)	0.162	0.0	0.944			
CHRNA3	rs6495309	T vs. C	Asian	3	1,865/1,983	Allelic	Fixed	0.770 (0.704–0.843)	<0.001	0.0	0.400	AAA	<0.001	Strong
CHRNA3	rs6495309	T vs. C	Asian	3	1,865/1,983	Dominant	Fixed	0.736 (0.642–0.843)	<0.001	0.0	0.749	AAA	<0.001	Strong
CHRNA3	rs6495309	T vs. C	Asian	3	1,865/1,983	Recessive	Fixed	0.680 (0.580–0.797)	<0.001	0.0	0.373	BAA	<0.001	Strong
CHRNA3	rs8042374	G vs. A	Caucasian	3	8,501/2,920	Allelic	Random	0.814 (0.703–0.941)	0.006	67.7	0.045	ACC	0.005	Moderate
CHRNA3	rs8042374	G vs. A	Caucasian	3	8,501/2,920	Dominant	Fixed	0.763 (0.697–0.834)	<0.001	47.3	0.150	ABC	<0.001	Moderate
CHRNA3	rs8042374	G vs. A	Caucasian	3	8,501/2,920	Recessive	Random	0.715 (0.503–1.016)	0.061	62.6	0.069			
CHRNA3	rs938682	A vs. G	Overall	3	4,958/2,575	Allelic	Fixed	1.239 (1.131–1.357)	<0.001	0.0	0.384	AAC	<0.001	Moderate
CHRNA3	rs938682	A vs. G	Overall	3	4,958/2,575	Dominant	Random	1.233 (0.829–1.834)	0.301	61.8	0.073			
CHRNA3	rs938682	A vs. G	Overall	3	4,958/2,575	Recessive	Fixed	1.295 (1.158–1.447)	<0.001	0.0	0.932	AAA	<0.001	Strong
CHRNA5	rs16969968	A vs. G	Overall	14	22,794/82,907	Allelic	Fixed	1.293 (1.260–1.328)	<0.001	25.1	0.183	ABA	<0.001	Strong
CHRNA5	rs16969968	A vs. G	Overall	14	22,794/82,907	Dominant	Fixed	1.374 (1.324–1.426)	<0.001	34.4	0.100	ABA	<0.001	Strong
CHRNA5	rs16969968	A vs. G	Overall	14	22,794/82,907	Recessive	Fixed	1.445 (1.372–1.522)	<0.001	0.0	0.568	AAA	<0.001	Strong
CHRNA5	rs16969968	A vs. G	Caucasian	12	21,035/80,599	Allelic	Fixed	1.298 (1.264–1.333)	<0.001	0.0	0.575	AAC	<0.001	Moderate
CHRNA5	rs16969968	A vs. G	Caucasian	12	21,035/80,599	Dominant	Fixed	1.384 (1.333–1.437)	<0.001	0.0	0.495	AAA	<0.001	Strong
CHRNA5	rs16969968	A vs. G	Caucasian	12	21,035/80,599	Recessive	Fixed	1.447 (1.374–1.524)	<0.001	0.0	0.540	AAC	<0.001	Moderate
CHRNA5	rs588765	C vs. T	Caucasian	4	5,851/7,321	Allelic	Fixed	1.124 (1.069–1.182)	<0.001	0.0	0.890	AAC	<0.001	Moderate
CHRNA5	rs588765	C vs. T	Caucasian	4	5,851/7,321	Dominant	Fixed	1.122 (1.020–1.234)	0.018	0.0	0.724	AAC	0.018	Moderate
CHRNA5	rs588765	C vs. T	Caucasian	4	5,851/7,321	Recessive	Fixed	1.192 (1.109–1.280)	<0.001	0.0	0.898	AAA	<0.001	Strong

OR, odds ratio; A, adenine; T, thymine; G, guanine; C, cytosine.

a Minor alleles vs. major alleles (reference).

b Strength of epidemiological evidence based on the Venice criteria.

c FPRP values at prior probability of 0.05 at power OR of 1.5, and the FPRP level of noteworthiness is 0.20.

d Degree of epidemiological credibility based on the combination of results from Venice guidelines and FPRP tests.

#### 3.2.1 COPD

We identified a nominally statistical association between *CHRNA3* rs1051730 and COPD risk under the allelic and dominant models in all populations (allelic model: OR = 1.630, 95% CI = 1.293–2.054, *p* < 0.001; dominant model: OR = 1.662, 95% CI = 1.300–2.124, *p* < 0.001), a nominally significant association under the allelic and dominant models in Asians (allelic model: OR = 1.591, 95% CI = 1.204–2.103, *p* = 0.001; dominant model: OR = 1.625, 95% CI = 1.222–2.160, *p* = 0.001), and a null association under all three models in Caucasians. Regarding *CHRNA5* rs16969968, we found a nominally statistical association between SNP rs16969968 and COPD risk under the three genetic models in all populations (allelic model: OR = 1.307, 95% CI = 1.205–1.417, *p* < 0.001; dominant model: OR = 1.413, 95% CI = 1.268–1.573, *p* < 0.001; recessive model: OR = 1.370, 95% CI = 1.154–1.625, *p* < 0.001) and a nominal association under the allelic and dominant models in Asians (allelic model: OR = 1.591, 95% CI = 1.204–2.103, *p* = 0.001; dominant model: OR = 1.625, 95% CI = 1.222–2.160, *p* = 0.001). We discovered a null association between *CHRNA5* rs16969968 and Caucasians under the three genetic models. In addition, we found that SNP rs6495309 was statistically related to COPD risk under the allelic and dominant models in Asians (allelic model: OR = 0.830, 95% CI = 0.759–0.906, *p* < 0.001; dominant model: OR = 0.736, 95% CI = 0.644–0.842, *p* = 0.001).

#### 3.2.2 Lung cancer

We detected a nominally statistical association between *CHRNA3* rs1051730 and LC risk under the three models in all populations (allelic model: OR = 1.348, 95% CI = 1.276–1.424, *p* < 0.001; dominant model: OR = 1.446, 95% CI = 1.342–1.559, *p* < 0.001; recessive model: OR = 1.519, 95% CI = 1.356–1.700, *p* < 0.001) and a nominally statistical association under the allelic and dominant models in Asians (allelic model: OR = 2.280, 95% CI = 1.626–3.197, *p* < 0.001; dominant model: OR = 2.329, 95% CI = 1.649–3.291, *p* < 0.001). There was also a nominally significant association under the three models in Caucasians (allelic model: OR = 1.313, 95% CI = 1.240–1.390, *p* < 0.001; dominant model: OR = 1.389, 95% CI = 1.283–1.504, *p* < 0.001; recessive model: OR = 1.506, 95% CI = 1.344–1.688, *p* < 0.001). For *CHRNA3* rs12914385, we presented no statistical association between rs12914385 and LC risk under the three models in all populations but a nominally conspicuous relationship with LC risk under the three models in Caucasians (allelic model: OR = 1.264, 95% CI = 1.053–1.517, *p* = 0.012; dominant model: OR = 1.458, 95% CI = 1.331–1.596, *p* = 0.014; recessive model: OR = 1.449, 95% CI = 1.134–1.851, *p* = 0.003) and a null relationship between *CHRNA3* rs12914385 and LC risk under the three models in Asians.

Regarding *CHRNA3* rs578776, we identified a nominally statistical relationship between SNP rs578776 and LC risk under two models in all populations, including Asians and Caucasians (allelic model: OR = 0.868, 95% CI = 0.773–0.976, *p* = 0.018; dominant model: OR = 0.841, 95% CI = 0.722–0.979*, p* = 0.026). We also demonstrated that *CHRNA3* rs6495309 likely had a statistical relationship with LC risk under the three models in Asians (allelic model: OR = 0.770, 95% CI = 0.704–0.843, *p* < 0.001; dominant model: OR = 0.736, 95% CI = 0.642–0.843, *p* < 0.001; recessive model: OR = 0.680, 95% CI = 0.580–0.797, *p* < 0.001). For *CHRNA3* rs8042374, we found a nominally statistical association between SNP rs8042374 and LC risk under two models in Caucasians (allelic model: OR = 0.814, 95% CI = 0.703–0.941, *p* = 0.006; dominant model: OR = 0.763, 95% CI = 0.697–0.834, *p* < 0.001). For *CHRNA3* rs938682, we detected a nominally statistical association between SNP rs938682 and LC risk under two models in all populations (allelic model: OR = 1.239, 95% CI = 1.131–1.357, *p* < 0.001; recessive model: OR = 1.295, 95% CI = 1.158–1.447, *p* < 0.001). In addition, *CHRNA3* rs3743073 was revealed to have no statistical relationship with LC risk under the three models in all populations, though this finding was not verified in Asians or Caucasians.

A nominally statistical association between *CHRNA5* rs16969968 and LC risk was shown under the three models in all populations (allelic model: OR = 1.293, 95% CI = 1.260–1.328, *p* < 0.001; dominant model: OR = 1.374, 95% CI = 1.324–1.426, *p* < 0.001; recessive model: OR = 1.445, 95% CI = 1.372–1.522, *p* < 0.001), and a nominally statistical association between *CHRNA5* rs16969968 and LC risk was identified under the three models in Caucasians (allelic model: OR = 1.293, 95% CI = 1.264–1.333, *p* < 0.001; dominant model: OR = 1.384, 95% CI = 1.333–1.437, *p* < 0.001; recessive model: OR = 1.447, 95% CI = 1.374–1.524, *p* < 0.001). In addition, a nominally statistical association was identified between SNP rs588765 and LC risk under the three models in Caucasians (allelic model: OR = 1.124, 95% CI = 1.069–1.182, *p* < 0.001; dominant model: OR = 1.122, 95% CI = 1.020–1.234, *p* = 0.018; recessive model: OR = 1.192, 95% CI = 1.109–1.280, *p* < 0.001).

Moreover, our study also found that some SNPs had no association with risk of disease. For example, our study found that rs12914385 in *CHRNA3* gene had a non-significant association with risk of LC in all populations under any genetic model, but had significant associations in Caucasians. rs12914385 in *CHRNA3* gene had no association with risk of LC in all populations.

### 3.3 Cumulative evidence of association

We initially used the Venice criteria to assess the cumulative epidemiological evidence for the eight SNPs that showed significant relationships to LC or COPD risk. More information on this evidence is listed in **Supplementary Table S4** and **Table 1**. For the Venice criteria test, we first assigned 30 A grades, 10 B grades, and 0 C grades to further assess the authenticity of evidence according to the amount of evidence. We then assigned 31 A grades, 5 B grades, and 4 C grades to evaluate their replication. Finally, we assigned 31 A grades, 0 B grades, and 9 C grades to assess protection from bias. Ultimately, the relationship to COPD risk could be rated according to three groups: strong (included *CHRNA3* rs6495309 under the dominant model in Asians), moderate (reflected eight associations, including *CHRNA3* rs1051730 under the allelic and dominant models in all populations and the allelic and dominant models in Asians, *CHRNA3* rs6495309 under the allelic model in Asians and *CHRNA5* rs16969968 under the recessive model in all populations, and the allelic and dominant models in Asians), and weak (represented two associations, including *CHRNA5* rs16969968 under the allelic and dominant models in all populations).

In terms of LC risk, the strong group was rated for 13 associations (including *CHRNA3* rs1051730 under the dominant and recessive models in all populations and under all three models in Caucasians, *CHRNA3* rs578776 under the allelic and dominant models in all populations, *CHRNA3* rs6495309 under the allelic and dominant models in Asians, *CHRNA3* rs938682 under the recessive model in all populations, *CHRNA5* rs16969968 under the recessive model in all populations and under the dominant model in Caucasians, and *CHRNA5* rs588765 under the recessive model in Caucasians); the moderate group was rated for 6 associations (including *CHRNA3* rs1051730 under the allelic model in all populations and in Asians, *CHRNA3* rs6495309 under the recessive model in Asians, and *CHRNA5* rs16969968 under the allelic and dominant models in all populations); and the weak group was rated for 10 associations (including *CHRNA3* rs12914385 under all three models in Caucasians, *CHRNA3* rs8042374 under the allelic and dominant models in Caucasians, *CHRNA3* rs938682 under the allelic model in all populations, *CHRNA5* rs16969968 under the allelic and recessive models in Caucasians, and *CHRNA5* rs588765 under the allelic and dominant models in Caucasians). In addition, we calculated the FPRP values of these nominally statistical associations to evaluate the probability of a precisely significant relationship between SNPs and LC or COPD risk. Of note, the *p*-values of the FPRP assay above all nominally significant associations between SNPs and LC or COPD risk were less than 0.05.

Finally, strong evidence was assigned to six variants with LC or COPD risk (28 significant associations). A strong association was identified between *CHRNA3* rs1051730 and COPD risk under the allelic and dominant models in all populations and in Asians; between *CHRNA3* rs6495309 and COPD risk under the allelic and dominant models in Asians; between *CHRNA5* rs16969968 and COPD risk under the recessive model in all populations and under the allelic and dominant models in Asians; between *CHRNA3* rs1051730 and LC risk under all three models in all populations and in Caucasians and under the allelic and dominant models in Asians; between *CHRNA3* rs578776 and LC risk under the allelic and dominant models in all populations; between *CHRNA3* rs6495309 and LC risk under all three models in Asians; between *CHRNA3* rs938682 and LC risk under the recessive model in all populations; between *CHRNA5* rs16969968 and LC risk under all three models in all populations and under the dominant model in Caucasians; and between *CHRNA5* rs588765 and LC risk under the recessive model in Caucasians. Moderate evidence was assigned to five SNPs (12 associations) with risk of LC or COPD. A moderate relationship was identified between *CHRNA5* rs16969968 and COPD risk under the allelic and dominant models in all populations; between *CHRNA3* rs12914385 and LC risk under all three models in Caucasians; between *CHRNA3* rs8042374 and LC risk under the allelic and dominant models in Caucasians; between *CHRNA3* rs938682 and LC risk under the allelic model in all populations; between *CHRNA5* rs16969968 and LC risk under the allelic and dominant models in Caucasians; and between *CHRNA5* rs588765 and LC risk under the allelic and dominant models in Caucasians.

### 3.4 Heterogeneity, bias, and sensitivity analyses

We performed assessments of heterogeneity, bias, and sensitivity analyses (**Supplementary Table S4** and **Table 1**). For nominally significant associations between SNPs and COPD risk, low heterogeneity was found for associations of *CHRNA3* rs1051730 in all populations (allelic model: *I* ^2^ = 0.0%, *p* = 0.897; dominant model: *I* ^2^ = 0.0%, *p* = 0.877) and in Asians (allelic model: *I* ^2^ = 0.0%, *p* = 0.780; dominant model: *I* ^2^ = 0.0%, *p* = 0.748); for associations of *CHRNA3* rs6495309 in Asians (dominant model: *I* ^2^ = 0.0%, *p* = 0.892); and for associations of *CHRNA5* rs16969968 in all populations (allelic model: *I* ^2^ = 0.0%, *p* = 0.471; dominant model: *I* ^2^ = 0.0%, *p* = 0.687; recessive model: *I* ^2^ = 0.0%, *p* = 0.713) and in Asians (allelic model: *I* ^2^ = 0.0%, *p* = 0.780; dominant model: *I* ^2^ = 0.0%, *p* = 0.748). Moderate heterogeneity was detected only for associations of *CHRNA3* rs6495309 (allelic model: *I* ^2^ = 25.9%, *p* = 0.259). In addition, we found little proof of publication bias for nominally significant associations between SNPs and COPD risk (*p* > 0.10), except in the case of *CHRNA5* rs16969968 under the allelic and dominant models in all populations (*p* < 0.10). Furthermore, we evaluated the robustness of these nominally significant associations by performing a sensitivity analysis that removed single studies (or datasets). The removal of studies that had been published first or studies deviating from HWE in COPD control groups did not alter the summary ORs. We did not test the excess of significant findings due to the unavailability of genotype amounts in most studies.

For nominally significant associations between SNPs and LC risk, the following associations were considered to have low heterogeneity: *CHRNA3* rs1051730 in all populations (dominant model: *I* ^2^ = 10.0%, *p* = 0.349; recessive model: *I* ^2^ = 0.0%, *p* = 0.582), in Asians (allelic model: *I* ^2^ = 0.0%, *p* = 0.978; dominant model: *I* ^2^ = 0.0%, *p* = 0.982), and in Caucasians (allelic model: *I* ^2^ = 0.0%, *p* = 0.611; dominant model: *I* ^2^ = 0.0%, *p* = 0.886; recessive model: *I* ^2^ = 8.0%, *p* = 0.365); *CHRNA3* rs578776 in all populations (allelic model: *I* ^2^ = 0.0%, *p* = 0.908; dominant model: *I* ^2^ = 0.0%, *p* = 0.937); *CHRNA3* rs6495309 in Asians (allelic model: *I* ^2^ = 0.0%, *p* = 0.400; dominant model: *I* ^2^ = 0.0%, *p* = 0.749; recessive model: *I* ^2^ = 0.0%, *p* = 0.373); *CHRNA3* rs938682 in all populations (allelic model: *I* ^2^ = 0.0%, *p* = 0.384; recessive model: *I* ^2^ = 0.0%, *p* = 0.932); *CHRNA5* rs16969968 in all populations (recessive model: *I* ^2^ = 0.0%, *p* = 0.568) and in Caucasians (allelic model: *I* ^2^ = 0.0%, *p* = 0.575; dominant model: *I* ^2^ = 0.0%, *p* = 0.495; recessive model: *I* ^2^ = 0.0%, *p* = 0.540); and *CHRNA5* rs588765 in Caucasians (allelic model: *I* ^2^ = 0.0%, *p* = 0.890; dominant model: *I* ^2^ = 0.0%, *p* = 0.724; recessive model: *I* ^2^ = 0.0%, *p* = 0.898). Four associations were found to have moderate heterogeneity: *CHRNA3* rs1051730 in all populations (allelic model: *I* ^2^ = 37.0%, *p* = 0.103); *CHRNA3* rs8042374 in Caucasians (dominant model: *I* ^2^ = 47.3%, *p* = 0.150); and *CHRNA5* rs16969968 in all populations (allelic model: *I* ^2^ = 25.1%, *p* = 0.183; dominant model: *I* ^2^ = 34.4%, *p* = 0.100). Another four associations were found to have large heterogeneity: *CHRNA3* rs12914385 in Caucasians (allelic model: *I* ^2^ = 84.4%, *p* = 0.002; dominant model: *I* ^2^ = 81.5%, *p* = 0.004; recessive model: *I* ^2^ = 66.5%, *p* = 0.051) and *CHRNA3* rs8042374 in Caucasians (allelic model: *I* ^2^ = 67.7%, *p* = 0.045).

As with the associations connected to COPD risk, we also performed a publication bias test. The test showed little confidence for nominally significant associations between SNPs and LC risk (*p* > 0.10), except in *CHRNA3* rs8042374 under the allelic and dominant models in Caucasians, *CHRNA3* rs938682 under the allelic model in all populations, *CHRNA5* rs16969968 under the allelic and recessive models in Caucasians, and *CHRNA5* rs588765 under the allelic and dominant models in Caucasians (*p* < 0.10). Regarding sensitivity analysis, the summary ORs were not modified by removing studies that had been published first or studies deviating from HWE in LC control groups, except *CHRNA5* rs588765 under the dominant model in Caucasians due to the removal of a study that had been published first. We did not test the excess of significant findings due to the unavailability of genotype amounts in most studies.

### 3.5 Functional annotation

**Figure 2:**
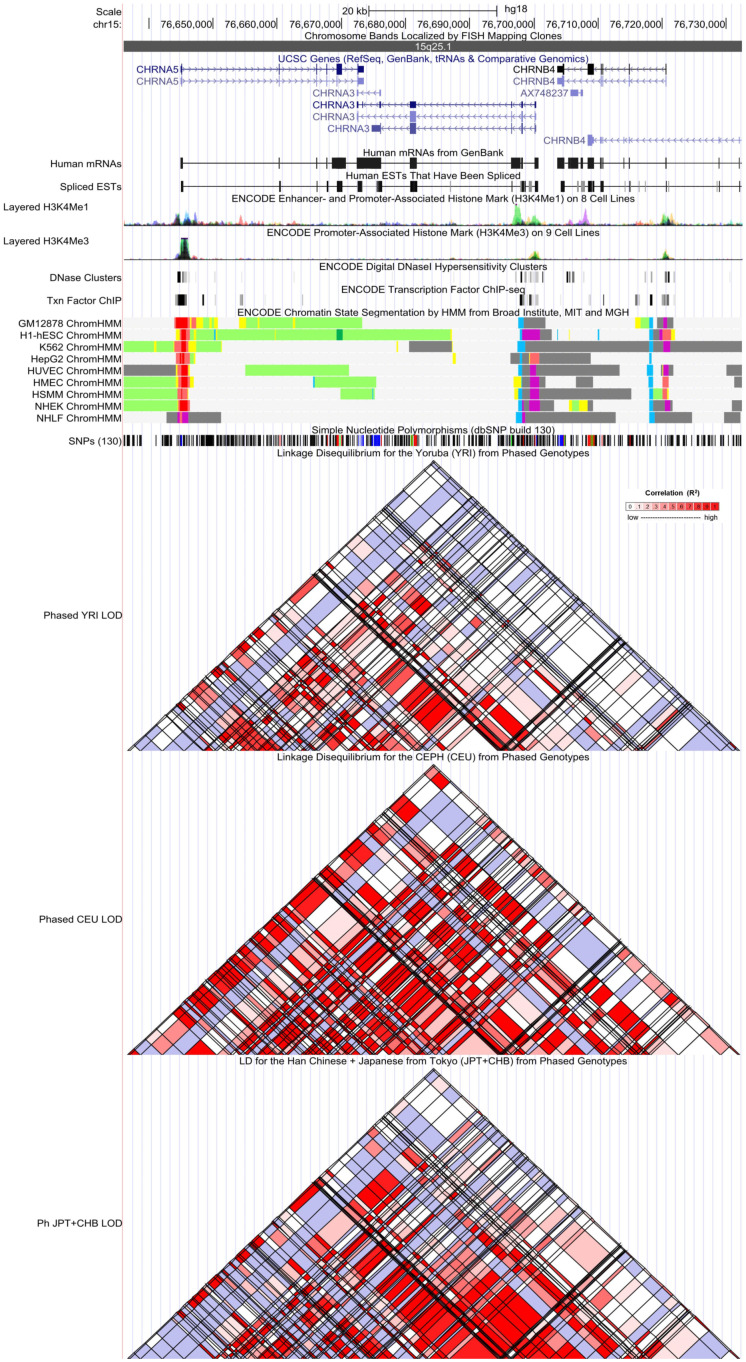
Evidence from the Encyclopedia of DNA Elements (ENCODE) data for the regulatory function of variants in 15q25.1 using the UCSC Genome Browser. The plot represents 15q25.1 within a 20-kb window centered on the CHRNA5–CHRNA3 gene region. Tracks (from top to bottom) in each of the plots are Genome Base Position, Chromosome Bands, UCSC Genes, Human messenger RNAs from GenBank, Human expressed sequence tag (ESTs) That Have Been Spliced, ENCODE Enhancer and Promoter-Associated Histone Mark (H3K4Me1) on 8 Cell Lines, ENCODE Promoter-Associated Histone Mark (H3K4Me3) on 9 Cell Lines, ENCODE Digital DNaseI Hypersensitivity Clusters, ENCODE Transcription Factor ChIP-seq, ENCODE Chromatin State Segmentation by Hidden Markov Model (HMM) from Broad Institute (bright red, active promoter; light red, weak promoter; purple, inactive/poised promoter; orange, strong enhancer; yellow, weak/poised enhancer; blue, insulator; dark green, transcriptional transition/elongation; light green, weak transcribed; gray, polycomb-repressed; light gray, heterochromatin/low signal/repetitive/copy number variation), Simple Nucleotide Polymorphisms (dbSNP build 130), Linkage Disequilibrium for the Yoruba (YRI) from Phased Genotypes, Linkage Disequilibrium for the CEPH (CEU) from Phased Genotypes, and LD for the Han Chinese + Japanese from Tokyo (JPT+CHB) from Phased Genotypes.

By referring to the data gained from the ENCODE tool, HaploReg v. 4.1, we analyzed the functional roles of the six variants strongly associated with LC or COPD risk (**Table 2**). The results showed that rs938682 and rs588765 mapped to intronic regions, rs578776 mapped to 3’UTR, rs1051730 was annotated as a synonymous variant, and rs16969968 was annotated as missense. Six SNPs could be considered expression quantitative trait loci (eQTLs) for numerous genes in various tissue types, six SNPs could be situated in the histone modification regions of enhancers, four SNPs could be located in promoters, and one SNP could be found in sites exhibiting DNase I hypersensitivity. We also found that four SNPs (rs1051730, rs6495309, rs578776, and rs938682) may be involved in transcriptional regulatory element activity. Moreover, linkage disequilibrium (LD) plots showed that regions delegated by significant SNPs had distinct genetic structures among European, Asian, and African ancestries (**Figure 2**). The Genotype-Tissue Expression Project revealed that rs1051730, rs16969968, and rs588765 are eQTLs for *CHRNA3* and *CHRNA5*. In brief, while rs1051730, rs16969968, and rs588765 are associated with a decrease of *CHRNA3* and *CHRNA5* gene expression in lung tissue, rs6495309, rs6495309, and rs938682 are associated with an increase in *IREB2* gene expression in lung tissue (**Supplementary Table 5**).

**Table 2:** Summary of functional annotations for six SNPs in CHRNA genes with diseases risk (strong epidemiological credibility).

Variant	Gene	Position^a^	Annotation	Promoter histone marks^b^	Enhancer histone marks^c^	DNase^d^	Proteins bound^e^	Motifs changed^f^
rs1051730	CHRNA3	78601997	Synonymous	LNG, SPLN	SPLN			AP-2, Foxl1, Foxo
rs6495309	CHRNA3	78622903		THYM	4 tissues	THYM		7 altered motifs
rs578776	CHRNA3	78596058	3'-UTR		ESDR			Hdx, Pou1f1
rs938682	CHRNA3	78604205	Intronic	ESDR, BLD, CRVX	ESC, ESDR, HRT			6 altered motifs
rs16969968	CHRNA5	78590583	Missense		ESC, IPSC			
rs588765	CHRNA5	78573083	Intronic	IPSC, HRT	ESC, LNG			

a The chromosome position is based on NCBI Build 37.

b Histone modification of H3K4me1 and H3K27ac (tissue types: if >3, only the number is included).

c Histone modification of H3K4me3 (tissue types: if >3, only the number is included).

d Levels of DNase I hypersensitivity (tissue types: if >3, only the number is included).

e Alteration in transcription factor binding (disruptions: if >3, only the number is included).

f Alteration in regulatory motif (disruptions: if >3, only the number is included).

## 4. Discussion

Although numerous existing studies have confirmed potential associations between *CHRNA* SNPs and LC or COPD risk, their results are inconsistent and controversial. To our knowledge, this is the first study to comprehensively elucidate whether the studied variants of *CHRNA* genes are related to the risk of cancerous or non-cancerous diseases and to evaluate the credibility of significant epidemiological evidence using the Venice criteria and FPRP tests. This meta-analysis assessed 29 papers with 70,960 cases and 124,838 controls. The study also evaluated the associations between nine SNPs and the risk of LC or COPD. Among these SNPs, eight were found to be statistically related to the risk of LC or COPD. In addition, two approaches (the Venice guidelines and FPRP tests) were used for the first time to assess these significant associations. The cumulative evidence for associations between six variants and LC or COPD risk was shown to be strong (28 significant associations: *CHRNA3* rs1051730, *CHRNA3* rs6495309, and *CHRNA5* rs16969968 with COPD risk and *CHRNA3* rs1051730, *CHRNA3* rs578776, *CHRNA3* rs6495309, *CHRNA3* rs938682, *CHRNA5* rs16969968, and *CHRNA5* rs588765 with LC risk), and the cumulative evidence for associations between five SNPs (12 associations) and LC or COPD risk was moderate. The current study also constructed functional annotations for the six SNPs with strong evidence using information from the ENCODE project and other public databases, revealing that these mutations may lie in several putative regulatory areas. In brief, this research provided comprehensive epidemiological evidence that familiar variants in *CHRNA* genes show an association with a predisposition to LC or COPD.

The *CHRNA3* gene (Gene ID: 1136) and *CHRNA5* gene (Gene ID: 1138), which are located in chromosome 15q25.1, were found to be related to the risk of LC and COPD (14, 15, 18). Many published papers, including GWAS, have demonstrated a significant relationship between *CHRNA* and COPD risk (15, 18). Some studies have indicated that the nicotine receptors expressed in lung epithelial cells can facilitate cancer cell proliferation and metastases (11–13). In the present study, six variants were shown to present with strong cumulative evidence in their associations with LC or COPD risk (*CHRNA3* rs1051730, *CHRNA3* rs6495309, and *CHRNA5* rs16969968 with COPD risk, and *CHRNA3* rs1051730, *CHRNA3* rs578776, *CHRNA3* rs6495309, *CHRNA3* rs938682, *CHRNA5* rs16969968, and *CHRNA5* rs588765 with LC risk).

For the evaluation of COPD, three SNPs showed strong associations with the risk of COPD. Phase 3 of the 1000 Genomes Project (**Supplementary Table 6**) suggested that CHRNA3 rs1051730 is uncorrelated with CHRNA3 rs6495309 in both Asians and Africans (*r* ^2^ < 0.05), and these SNPs showed weak LD in Europeans (*r* ^2^ = 0.1751) (40). Furthermore, *CHRNA5* rs16969968 was uncorrelated with *CHRNA3* rs6495309 in both Asians and Africans (*r* ^2^ < 0.05), and these SNPs were weak in Europeans (*r* ^2^ = 0.1729). According to the results, different causal mutations and functional mechanisms may exist in the relationships between COPD risk and mutations in the *CHRNA3* and/or *CHRNA5* genes. Moreover, while *CHRNA5* rs16969968 showed strong LD with *CHRNA3* rs1051730 in both Asians and Europeans (*r* ^2^ > 0.9), it showed weak LD in Africans (*r* ^2^ = 0.2520). Based on the results, the functional mechanisms of the two variants may change among different ethnic groups and partially answer to ethnic differences among variants related to disease, such as COPD risk.

For the assessment of LC, six SNPs were strongly related to LC risk (rs1051730, rs578776, rs6495309, and rs938682 in the *CHRNA3* gene and rs16969968 and rs588765 in the *CHRNA5* gene). Phase 3 of the 1000 Genomes Project (**Supplementary Table 6**) suggested that CHRNA3 rs6495309 showed a strong LD with CHRNA3 rs938682 in both Asians and Europeans (*r* ^2^ > 0.8) and a moderate LD in Africans (*r* ^2^ = 0.5143). While CHRNA3 rs578776 showed a strong LD with CHRNA3 rs938682 in Europeans (*r* ^2^ > 0.8), these SNPs showed weak LD in both Asians (*r* ^2^ = 0.2185) and Africans (*r* ^2^ = 0.2991). Furthermore, CHRNA3 rs6495309 showed moderate LD with CHRNA3 rs578776 in Europeans (*r* ^2^ = 0.7556), but these SNPs showed weak LD in both Asians (*r* ^2^ = 0.1698) and Africans (*r* ^2^ = 0.1289). Finally, although CHRNA5 rs16969968 showed moderate LD with CHRNA5 rs588765 in Europeans (*r* ^2^ = 0.3601), these SNPs were uncorrelated in both Asians and Africans (*r* ^2^ < 0.05). Based on the results, the functional mechanisms of the three variants related to the risk of LC may be different across ethnic groups and may partially answer the ethnic differences of some variants related to disease. Finally, rs1051730 showed weak LD or was uncorrelated with three SNPs (rs6495309, rs578776, and rs938682), indicating that different causal mutations and functional mechanisms may exist in the relationships between LC risk and *CHRNA3* gene mutations.

Current evidence has indicated that both *CHRNA3* rs1051730 and *CHRNA5* rs16969968 have excellent responses to nicotinic agonists *in vitro* (41). When compared to smokers without these two SNPs, these polymorphisms are generally present in heavy smokers who have higher levels of nitrosamines and other derivatives due to the combustion of tobacco, which can trigger an inflammatory response to COPD and elevated cellular proliferation in lung tissue, resulting in the development of LC or COPD (14). *CHRNA3* rs6495309 can change the binding ability of the transcriptional factor *Oct-1*, which has been shown to repress gene transcription, leading to alterations in *CHRNA3* RNA expression. This influences the ability of cells to progress into apoptosis, thereby impacting LC risk (17). Interestingly, the rs6495309 T allele has a decreased susceptibility to COPD due to reduced promoter activity, which diminishes *CHRNA3* expression and the inflammatory response to smoking exposure (25). A previous study showed that the rs578776 A allele could reduce the risk of nicotine dependence and the risk of LC in Caucasians (42). Moreover, the SNP rs588765 was reported to be linked to changes in *CHRNA5* mRNA expression in lung tissue and to show a strong relationship with nicotine dependence (23, 43). In European, Asian, and African populations, four variants in the *CHRNA3* gene were shown to be uncorrelated or show weak LD with two mutations in the *CHRNA5* gene. According to the results, different causal mutants and functional mechanisms exist in the relationships between mutations in the *CHRNA3* and *CHRNA5* genes and LC susceptibility.

Moreover, this study showed that some SNPs had no association with LC risk. Briefly, our study analyzing the same SNP from different groups yielded inconsistent results due to the selection of association models, ethnicity, and variations in sample size. For the inconsistent results in different genetic models, the existence of different genetic backgrounds such as age and gender of patients, subtypes of cancers, and environmental factors were not taken into consideration and may present as sources of variation in the result. The minor allele frequency of SNP had differences among different races, and studies with smaller sample sizes had low statistical power, which may explain why these associations produced inconsistent results in different ethnicities.

While this study provides the largest sample and a comprehensive evaluation of variants related to the risk of cancerous and non-cancerous diseases, it contains several limitations. First, although comprehensive research was conducted on databases, some publications may have been missed, and certain papers may have lacked sufficient data, such as the genotype amount, resulting in an incomplete assessment of other malignancies and non-cancerous diseases. Second, sufficient data could not be provided for assessments of the interactions between different variants and the adjusted effect of environmental factors, including smoking and *H. pylori* infection. Third, a detailed subgroup analysis of cancer types ascribed to the heterogeneity of cancer typing among eligible studies was not completed. Fourth, the excess of significant findings was not alternatively evaluated due to insufficient data. Finally, some of the significant findings were identified with moderate credibility. Because this was partially due to the small sample of subgroups related to ethnicity under different genetic models, studies with sufficient subgroups are recommended to validate the current research’s findings.

In this extensively updated meta-analysis, eight SNPs were proven to be significantly related to LC and COPD risk; of these, six variants were graded to show strong cumulative evidence for LC or COPD predisposition (*CHRNA3* rs1051730, *CHRNA3* rs6495309, and *CHRNA5* rs16969968 with COPD risk and *CHRNA3* rs1051730, *CHRNA3* rs578776, *CHRNA3* rs6495309, *CHRNA3* rs938682, *CHRNA5* rs16969968, and *CHRNA5* rs588765 with LC risk), and five SNPs were graded to show moderate cumulative evidence for LC or COPD risk. This study also provides a basis for further understanding of the genetic predisposition of LC and COPD susceptibility. Our findings could inspire further studies to elucidate the cause of LC and COPD and may lead to the development of screening and prevention strategies for clinical management.

## Data availability statement

The original contributions presented in the study are included in the article/**Supplementary Material**. Further inquiries can be directed to the corresponding author.

## Author contributions

LY and HC designed this work. LY and XL integrated and analyzed the data. LY and HC wrote this manuscript. LY, CZ, XL, TL, ZY and CJ finished the related tables and figures. LY and HC edited and revised the manuscript. All authors contributed to the article and approved the submitted version.

## Funding

This study was supported by funding from the Chongqing Natural Science Foundation (grant no. cstc2020jcyj-msxmX0257).

## Conflict of interest

The authors declare that the research was conducted in the absence of any commercial or financial relationships that could be construed as a potential conflict of interest.

## Publisher’s note

All claims expressed in this article are solely those of the authors and do not necessarily represent those of their affiliated organizations, or those of the publisher, the editors and the reviewers. Any product that may be evaluated in this article, or claim that may be made by its manufacturer, is not guaranteed or endorsed by the publisher.
