# Supplementary material for: Epidemiological evidence for associations between variants in CHRNA genes and risk of lung cancer and chronic obstructive pulmonary disease
Source: Front Oncol. 2022 Oct 6;12:1001864. doi: 10.3389/fonc.2022.1001864 (PMC9582127; doi:10.3389/fonc.2022.1001864)
Supplement: Supplementary file 1 [file DataSheet_1.pdf]

Supplementary Materials to:

Epidemiological Evidence for Associations Between Variants in CHRNA Genes and Risk of Lung Cancer and Chronic Obstructive Pulmonary Disease

Lei Yang[1#], Zelin Yang[1#], Chunjian Zuo[2], Xiaolong Lv[1], Tianyu Liu[1], Chenhao Jia[1], Huanwen Chen[1*]

[1]Department of Cardiothoracic Surgery, The First Affiliated Hospital of Chongqing Medical University, Chongqing 400010, China.

[2]Department of Thoracic Surgery, Army Medical Center of PLA, No.10, Daping Changjiang Branch Road, Yuzhong District, Chongqing 400042, China.

[#] These authors have contributed equally to this work.

[*]**Corresponding author:** Huanwen Chen, M.D., Ph.D.

Department of Cardiothoracic Surgery

The First Affiliated Hospital of Chongqing Medical University, Chongqing 400010, China.

Mailing address: No.1,Youyi Road, Yuzhong District, Chongqing 400010, China.

Phone: 86-8901-1132; Fax: 86-8901-1132;

Email address: coolstarchw9527@163.com

Contents

Supplementary Notes

Supporting information to Notes for Venice Criteria.

Supplementary Tables

Supplementary Table S1. PRISMA checklist items was presented in our study.

Supplementary Table S2. Genetic model with the complete data structure of genetic polymorphism study.

Supplementary Table S3. Characteristics of the included articles.

Supplementary Table S4. Associations between variants in the *CHRNA* genes with cancer or noncancerous diseases risk.

Supplementary Table S5. Analyses of expression quantitative trait locus (eQTL) in significant variants associated with risk of disease.

Supplementary Table S6. Linkage Disequilibrium (r^2) among the significant variants in the *CHRNA* genes

Supporting information to Notes for Venice Criteria

We applied the Venice Criteria to evaluate the epidemiological credibility of significant associations identified by meta-analysis. Briefly, credibility was defined as strong, moderate, or weak, based on the grade of A, B, or C in three categories: amount of evidence, replication of the association, and protection from bias.

Amount of evidence

A: Large-scale evidence — minor genetic group (alleles or genotypes) in cases and controls > 1,000.

B: Moderate amount of evidence — minor genetic group in cases and controls between 100 and 1,000.

C: Little evidence — minor genetic group in cases and controls < 100.

Replication of association

A: Little between-study heterogeneity — $I^2 < 25\%$.

B: Moderate between-study heterogeneity — I^2 between 25% and 50%.

C: Large between-study heterogeneity — $I^2 > 50\%$.

Qualitative epidemiologic considerations about the presence of heterogeneity and potential explanation for heterogeneity would need to be taken into account in judging replication. It may be reasonable to grade as A on this criterion for associations with moderate or high heterogeneity with an extensive replication record such as associations identified by GWAS or large GWAS meta-analysis from collaborative studies.

Protection from bias

A: No observable bias and bias was unlikely to explain the presence of the association. B: No obvious bias may affect the presence of the association, but there is considerable missing information on the identification of evidence. C: Bias is demonstrable or is likely to explain the presence of the association. The Venice criteria include an extensive checklist for sources of bias in different settings. The checklist has different considerations depending on whether the evidence comes from retrospective meta-analyses of published data or prospective GWAS and replication studies from collaborative consortia with harmonization of data collection and analysis.

General checks for bias that have been adopted for meta-analysis are: (1) Association lost with exclusion of first study; (2) Association lost with exclusion of studies deviated from HWE; (3) Small effect size of association (i.e., $0.87 < OR < 1.15$); (4) Evidence of publication bias ($p < 0.10$ in Begg's test); (5) Evidence of small-study effect ($p < 0.10$ in Egger's test); (6) Evidence is presented for an excess of individual studies with significant findings ($p < 0.10$ in significant bias test).

Supplementary Table S1: PRISMA checklist items was presented in our study

Section/topic	Items	Checklist item	Reported on page
**TITLE**			
Title	1	Identify the report as a systematic review, meta-analysis, or both.	1
**ABSTRACT**			
Structured summary	2	Provide a structured summary including, as applicable: background; objectives; data sources; study eligibility criteria, participants, and interventions; study appraisal and synthesis methods; results; limitations; conclusions and implications of key findings; systematic review registration number.	2
**INTRODUCTION**			
Rationale	3	Describe the rationale for the review in the context of what is already known.	1-2
Objectives	4	Provide an explicit statement of questions being addressed with reference to participants, interventions, comparisons, outcomes, and study design (PICOS).	2-3
**METHODS**			
Protocol and registration	5	Indicate if a review protocol exists, if and where it can be accessed (e.g., Web address), and, if available, provide registration information including registration number.	/
Eligibility criteria	6	Specify study characteristics (e.g., PICOS, length of follow-up) and report characteristics (e.g., years considered, language, publication status) used as criteria for eligibility, giving rationale.	4
Information sources	7	Describe all information sources (e.g., databases with dates of coverage, contact with study authors to identify additional studies) in the search and date last searched.	4

Section/topic	Items	Checklist item	Reported on page
Search	8	Present full electronic search strategy for at least one database, including any limits used, such that it could be repeated.	4
Study selection	9	State the process for selecting studies (i.e., screening, eligibility, included in systematic review, and, if applicable, included in the meta-analysis).	4
Data collection process	10	Describe method of data extraction from reports (e.g., piloted forms, independently, in duplicate) and any processes for obtaining and confirming data from investigators.	5
Data items	11	List and define all variables for which data were sought (e.g., PICOS, funding sources) and any assumptions and simplifications made.	5,6
Risk of bias in individual studies	12	Describe methods used for assessing risk of bias of individual studies (including specification of whether this was done at the study or outcome level), and how this information is to be used in any data synthesis.	5,6
Summary measures	13	State the principal summary measures (e.g., risk ratio, difference in means).	5,6
Synthesis of results	14	Describe the methods of handling data and combining results of studies, if done, including measures of consistency (e.g., I^2) for each meta-analysis.	5,6
Risk of bias across studies	15	Specify any assessment of risk of bias that may affect the cumulative evidence (e.g., publication bias, selective reporting within studies).	5,6
Additional analyses	16	Describe methods of additional analyses (e.g., sensitivity or subgroup analyses, meta-regression), if done, indicating which were pre-specified.	5,6
**RESULTS**			
Study selection	17	Give numbers of studies screened, assessed for eligibility, and included in the review, with reasons for exclusions at each stage, ideally with a flow diagram.	8

Section/topic	Items	Checklist item	Reported on page
Study characteristics	18	For each study, present characteristics for which data were extracted (e.g., study size, PICOS, follow-up period) and provide the citations.	8
Risk of bias within studies	19	Present data on risk of bias of each study and, if available, any outcome-level assessment (see Item 12).	/
Results of individual studies	20	For all outcomes considered (benefits or harms), present, for each study: (a) simple summary data for each intervention group and (b) effect estimates and confidence intervals, ideally with a forest plot.	8-12
Synthesis of results	21	Present results of each meta-analysis done, including confidence intervals and measures of consistency.	8-12
Risk of bias across studies	22	Present results of any assessment of risk of bias across studies (see Item 15).	13,14
Additional analysis	23	Give results of additional analyses, if done (e.g., sensitivity or subgroup analyses, meta-regression [see Item 16]).	13,14
**DISCUSSION**			
Summary of evidence	24	Summarize the main findings including the strength of evidence for each main outcome; consider their relevance to key groups (e.g., health care providers, users, and policy makers).	15-18
Limitations	25	Discuss limitations at study and outcome level (e.g., risk of bias), and at review level (e.g., incomplete retrieval of identified research, reporting bias).	18
Conclusions	26	Provide a general interpretation of the results in the context of other evidence, and implications for future research.	19
**FUNDING**			
Funding	27	Describe sources of funding for the systematic review and other support (e.g., supply of data); role of funder thes for the systematic review.	19

Supplementary Table S2: Genetic model with the complete data structure of genetic polymorphism study

Genotype amount			
Genotype type	AA	AB	BB
Case group	a_n	b_n	c_n
Control group	d_n	e_n	f_n

Abbreviation: AA:Wild homozygous; AB: Heterozygous mutant; BB: Mutant homozygous; n: indicating the Nth study.

Description: For a SNP, two alleles, A and B, could be presented. Specifically, A was considered as wild type, meanwhile, B was considered mutant type. Therefore, there may be three genotypes, AA, AB, BB, respectively, in population. Suppose there were there three genotypes of the subjects, we could assign a,b,c to AA, AB, BB in case group, and d,e,f to AA, AB, BB in control group, respectively. The table above could offer additional explanation.

In meta-analyses for SNPs, polygenic model was used to decrease probabilities of type I error. The following genetic models may be used in our study: 1) Allelic model (i.e. B vs A); 2) Dominant model (BB+BA vs AA); 3) Recessive model (BB vs BA+AA). In our meta-analyses, the mentioned three genetic models were all used to evaluate the associations between genetic variants in miRNAs and cancer risk.

Supplementary Table 3. Characteristics of the included articles.

Ref Number	First author, year	Disease	Disease Type	Country/region	Ethnicity	Study design	Gene name	variant	Allelic	Case	control	Sample size
1	Mimouni A, 2020	Lung cancer		Algeria	Caucasian	Case-control study	CHRNA5	rs16969968 G/A	A/ G	143	211	354
2	Sun Y, 2018	Lung cancer		China	Asian	AHBCCS	CHRNA3	rs6495309 C/T	C/T	306	306	612
3	Pérez-Morales R, 2018	Lung cancer		Mexico	Latin American	Case-control study	CHRNA3	rs1051730 G/A	G/A	74	192	266
3	Pérez-Morales R, 2018	COPD		Mexico	Latin American	Case-control study	CHRNA3	rs1051730 G/A	G/A	117	192	309
3	Pérez-Morales R, 2018	Lung cancer		Mexico	Latin American	Case-control study	CHRNA5	rs16969968 G/A	G/A	74	192	266
3	Pérez-Morales R, 2018	COPD		Mexico	Latin American	Case-control study	CHRNA5	rs16969968 G/A	G/A	117	192	309
4	Zhao Z, 2015	COPD		China	Asian	Case-control study	CHRNA3	rs1051730 G/A	G/A	600	610	1210
4	Zhao Z, 2015	COPD		China	Asian	Case-control study	CHRNA5	rs16969968 G/A	G/A	600	610	1210
4	Zhao Z, 2015	COPD		China	Asian	Case-control study	CHRNA3	rs1051730 G/A	G/A	413	420	833
4	Zhao Z, 2015	COPD		China	Asian	Case-control study	CHRNA5	rs16969968 G/A	G/A	413	420	833
5	Ren JH, 2013	Lung cancer		China	Asian	Case-control study	CHRNA3	rs1051730 G/A	G/A	210	200	410
6	Islam MS, 2013	Lung cancer		Bangladeshi	Caucasian	Case-control study	CHRNA5	rs16969968 G/A	G/A	106	116	222
7	Gabrielsen ME, 2013	Lung cancer		Norway	Caucasian	Case-control study	CHRNA5	rs16969968 G/A	G/A	459	55823	56282
7	Gabrielsen ME, 2013	COPD		Norway	Caucasian	Case-control study	CHRNA5	rs16969968 G/A	G/A	1063	5301	6364
8	Zhou H, 2012	COPD		China	Asian	Case-control study	CHRNA5	rs16969968 G/A	G/A	488	687	1175
8	Zhou H, 2012	COPD		China	Asian	Case-control study	CHRNA3	rs1051730 G/A	G/A	488	687	1175

9	Yang L, 2012	Lung cancer	China	Asian	AHBCCS	CHRNA3	rs6495309 C/T	C/T	1056	1061	2117
9	Yang L, 2012	Lung cancer	China	Asian	AHBCCS	CHRNA3	rs6495309 C/T	C/T	503	616	1119
9	Yang L, 2012	COPD	China	Asian	AHBCCS	CHRNA3	rs6495309 C/T	C/T	1025	1061	2086
9	Yang L, 2012	COPD	China	Asian	AHBCCS	CHRNA3	rs6495309 C/T	C/T	486	616	1102
10	Shen B, 2012	Lung cancer	China	Asian	AHBCCS	CHRNA3	rs3743073 G/T	G/T	600	600	1200
11	Li Z, 2012	Lung cancer	China	Asian	Case-control study	CHRNA3	rs578776 G/A	G/A	199	199	398
11	Li Z, 2012	Lung cancer	China	Asian	Case-control study	CHRNA3	rs938682 G/A	G/A	200	200	400
12	Wang Y, 2011	Lung cancer	UK	Caucasian	Case-control study	CHRNA3	rs12914385 C/T	C/T	4257	1460	5717
12	Wang Y, 2011	Lung cancer	UK	Caucasian	Case-control study	CHRNA3	rs8042374 A/G	A/G	4250	1460	5710
13	Sakoda LC, 2011	Lung cancer	unknown	Caucasian	Case-control study	CHRNA5	rs588765 T/C	T/C	745	1475	2220
13	Sakoda LC, 2011	Lung cancer	unknown	Caucasian	Case-control study	CHRNA5	rs16969968 G/A	G/A	743	1476	2219
13	Sakoda LC, 2011	Lung cancer	unknown	Caucasian	Case-control study	CHRNA3	rs578776 G/A	G/A	744	1473	2217
13	Sakoda LC, 2011	Lung cancer	unknown	Caucasian	Case-control study	CHRNA3	rs1051730 G/A	G/A	745	1475	2220
13	Sakoda LC, 2011	Lung cancer	unknown	Caucasian	Case-control study	CHRNA3	rs938682 G/A	G/A	739	1468	2207
14	Wang Y, 2010	Lung cancer	UK	Caucasian	Case-control study	CHRNA3	rs8042374 A/G	A/G	239	553	792
14	Wang Y, 2010	Lung cancer	UK	Caucasian	Case-control study	CHRNA3	rs12914385 C/T	C/T	239	553	792
15	Niu X, 2010	Lung cancer	China	Asian	Case-control study	CHRNA3	rs3743073 G/T	G/T	482	566	1048
16	Zienolddiny S, 2009	Lung cancer	Norway	Caucasian	Case-control study	CHRNA3	rs1051730 G/A	G/A	352	425	777
16	Zienolddiny S, 2009	Lung cancer	Norway	Caucasian	Case-control study	CHRNA5	rs16969968 G/A	G/A	357	426	783
17	Schwartz AG, 2009	Lung cancer	USA	Caucasian	Case-control study	CHRNA3	rs1051730 G/A	G/A	582	844	1426

17	Schwartz AG, 2009	Lung cancer	USA	Black	Case-control study	CHRNA3	rs1051730 G/A	G/A	385	432	817
18	Falvella FS, 2009	Lung cancer	Italy	Caucasian	APBCCS	CHRNA5	rs16969968 G/A	G/A	467	739	1206
19	Amos CI, 2008	Lung cancer	USA	Caucasian	Case-control study	CHRNA3	rs1051730 G/A	G/A	1153	1137	2290
19	Amos CI, 2008	Lung cancer	USA	Caucasian	Case-control study	CHRNA3	rs1051730 G/A	G/A	702	594	1296
19	Amos CI, 2008	Lung cancer	UK	Caucasian	Case-control study	CHRNA3	rs1051730 G/A	G/A	1830	956	2786
20	Broderick P, 2009	Lung cancer	UK	Caucasian	Case-control study	CHRNA3	rs12914385 C/T	C/T	4018	907	4925
20	Broderick P, 2009	Lung cancer	UK	Caucasian	Case-control study	CHRNA3	rs8042374 A/G	A/G	4012	907	4919
20	Broderick P, 2009	Lung cancer	UK	Caucasian	Case-control study	CHRNA3	rs938682 G/A	G/A	4019	907	4926
21	Jaworowska E, 2011	Lung cancer	Poland	Caucasian	Case-control study	CHRNA5	rs16969968 G/A	G/A	842	841	1683
22	Ji X, 2015	Lung cancer	Italy	Caucasian	Case-control study	CHRNA5	rs588765 T/C	T/C	1814	1953	3767
22	Ji X, 2015	Lung cancer	Italy	Caucasian	Case-control study	CHRNA5	rs16969968 G/A	G/A	1814	1953	3767
22	Ji X, 2015	Lung cancer	Europ	Caucasian	Case-control study	CHRNA5	rs588765 T/C	T/C	2983	3553	6536
22	Ji X, 2015	Lung cancer	Europ	Caucasian	Case-control study	CHRNA5	rs16969968 G/A	G/A	2983	3553	6536
23	Kohno T, 2011	Lung cancer	Japan	Asian	AHBCCS	CHRNA3	rs1051730 G/A	G/A	374	324	698
24	Lips EH, 2009	Lung cancer	Europ	Caucasian	Case-control study	CHRNA5	rs16969968 G/A	G/A	3306	3767	7073
25	Shiraishi K, 2009	Lung cancer	Japan	Asian	Case-control study	CHRNA3	rs1051730 G/A	G/A	1250	936	2186
26	Tekpli X, 2012	Lung cancer	Norway	Caucasian	Case-control study	CHRNA5	rs588765 T/C	T/C	309	340	649
26	Tekpli X, 2012	Lung cancer	Norway	Caucasian	Case-control study	CHRNA3	rs578776 G/A	G/A	302	337	639
26	Tekpli X, 2012	Lung cancer	Norway	Caucasian	Case-control study	CHRNA3	rs3743073 G/T	G/T	309	334	643
27	Truong T, 2010	Lung cancer	Mixed	Caucasian	Case-control study	CHRNA5	rs16969968 G/A	G/A	9378	11219	20597
27	Truong T, 2010	Lung cancer	Mixed	Asian	Case-control study	CHRNA5	rs16969968 G/A	G/A	1685	2116	3801

27	Truong T, 2010	Lung cancer	Mixed	Asian	Case-control study	CHRNA3	rs12914385 C/T	C/T	1523	1523	3046
28	Young RP, 2008	COPD	New Zealand	Caucasian	Case-control study	CHRNA5	rs16969968 G/A	G/A	445	475	920
28	Young RP, 2008	Lung cancer	New Zealand	Caucasian	Case-control study	CHRNA5	rs16969968 G/A	G/A	437	475	912
29	Lee JY, 2012	COPD	China	Asian	Case-control study	CHRNA3	rs6495309 C/T	C/T	406	394	800

Note: A, adenine; C, cytosine; G, guanine; T, thymine; AHBCCS: A hospital-based case control study; APBCCS: A population-based case control study. COPD: chronic obstructive pulmonary disease.

References:

1. Mimouni A, Rouleau E, Saulnier P, Marouani A, Abdelali ML, Filali T, Beddar L, Lakehal A, Hireche A, Boudersa A, Aissaoui M, Ramtani H, Bouhedjar K, Abdellouche D, Oudjehih M, Boudokhane I, Abadi N, Satta D. Association of TERT, OGG1, and CHRNA5 Polymorphisms and the Predisposition to Lung Cancer in Eastern Algeria. Pulm Med. 2020 Mar 20;2020:7649038. doi: 10.1155/2020/7649038. PMID: 32257438; PMCID: PMC7109590.
2. Sun Y, Li J, Zheng C, Zhou B. Study on polymorphisms in CHRNA5/CHRNA3/CHRNB4 gene cluster and the associated with the risk of non-small cell lung cancer. Oncotarget. 2017 Dec 20;9(2):2435-2444. doi: 10.18632/oncotarget.23459. PMID: 29416783; PMCID: PMC5788651.
3. Pérez-Morales R, González-Zamora A, González-Delgado MF, Calleros Rincón EY, Olivas Calderón EH, Martínez-Ramírez OC, Rubio J. CHRNA3 rs1051730 and CHRNA5 rs16969968 polymorphisms are associated with heavy smoking, lung cancer, and chronic obstructive pulmonary disease in a mexican population. Ann Hum Genet. 2018 Nov;82(6):415-424. doi: 10.1111/ahg.12264. Epub 2018 Jul 11. PMID: 29993116.
4. Zhao Z, Peng F, Zhou Y, Hu G, He H, He F, Zou W, Zhao Z, Li B, Ran P. Exon sequencing identifies a novel CHRNA3-CHRNA5-CHRNB4 variant that increases the risk for chronic obstructive pulmonary disease. Respirology. 2015 Jul;20(5):790-8. doi: 10.1111/resp.12539. Epub 2015 Apr 19. PMID: 25891420.
5. Ren JH, Jin M, He WS, Liu CW, Jiang S, Chen WH, Yang KY, Wu G, Zhang T. Association between CHRNA3 rs1051730 genotype and lung cancer risk in Chinese Han population: a case-control study. J Huazhong Univ Sci Technolog Med Sci. 2013 Dec;33(6):897-901. doi: 10.1007/s11596-013-1218-4. Epub 2013 Dec 13. PMID: 24337855.
6. Islam MS, Ahmed MU, Sayeed MS, Maruf AA, Mostofa AG, Hussain SM, Kabir Y, Daly AK, Hasnat A. Lung cancer risk in relation to nicotinic acetylcholine receptor, CYP2A6 and CYP1A1 genotypes in the Bangladeshi population. Clin Chim Acta. 2013 Feb 1;416:11-9. doi: 10.1016/j.cca.2012.11.011. Epub 2012 Nov 21. PMID: 23178447.
7. Gabrielsen ME, Romundstad P, Langhammer A, Krokan HE, Skorpen F. Association between a 15q25 gene variant, nicotine-related habits, lung cancer and COPD among 56,307 individuals from the HUNT study in Norway. Eur J Hum Genet. 2013 Nov;21(11):1293-9. doi: 10.1038/ejhg.2013.26. Epub 2013 Feb 27. PMID: 23443019; PMCID: PMC3798835.
8. Zhou H, Yang J, Li D, Xiao J, Wang B, Wang L, Ma C, Xu S, Ou X, Feng Y. Association of IREB2 and CHRNA3/5 polymorphisms with COPD and COPD-related phenotypes in a Chinese Han population. J Hum Genet. 2012 Nov 26;57(11):738-46. doi: 10.1038/jhg.2012.104. Epub 2012 Aug 23. PMID: 22914670.
9. Yang L, Qiu F, Lu X, Huang D, Ma G, Guo Y, Hu M, Zhou Y, Pan M, Tan Y, Zhong H, Ji W, Wei Q, Ran P, Zhong N, Zhou Y, Lu J. Functional polymorphisms of CHRNA3 predict risks of chronic obstructive pulmonary disease and lung cancer in Chinese. PLoS One. 2012;7(10):e46071. doi: 10.1371/journal.pone.0046071. Epub 2012 Oct 3. PMID: 23056235; PMCID: PMC3463594.
10. Shen B, Shi MQ, Zheng MQ, Hu SN, Chen J, Feng JF. Correlation between polymorphisms of nicotine acetylcholine acceptor subunit CHRNA3 and lung cancer susceptibility. Mol Med Rep. 2012 Dec;6(6):1389-92. doi: 10.3892/mmr.2012.1101. Epub 2012 Sep 26. PMID: 23023782.
11. Li Z, Bao S, Xu X, Bao Y, Zhang Y. Polymorphisms of CHRNA5-CHRNA3-CHRNB4 Gene Cluster and NSCLC Risk in Chinese Population. Transl Oncol. 2012 Dec;5(6):448-52. doi: 10.1593/tlo.12304. Epub 2012 Dec 1. PMID: 23397474; PMCID: PMC3567724.
12. Wang Y, Broderick P, Matakidou A, Eisen T, Houlston RS. Chromosome 15q25 (CHRNA3-CHRNA5) variation impacts indirectly on lung cancer risk. PLoS One. 2011 Apr 29;6(4):e19085. doi: 10.1371/journal.pone.0019085. PMID: 21559498; PMCID: PMC3084737.
13. Sakoda LC, Loomis MM, Doherty JA, Neuhouser ML, Barnett MJ, Thornquist MD, Weiss NS, Goodman GE, Chen C. Chromosome 15q24-25.1 variants, diet, and lung cancer susceptibility in cigarette smokers. Cancer Causes Control. 2011 Mar;22(3):449-61. doi: 10.1007/s10552-010-9716-1. Epub 2011 Jan 13. PMID: 21229299; PMCID: PMC3042523.
14. Wang Y, Broderick P, Matakidou A, Eisen T, Houlston RS. Role of 5p15.33 (TERT-CLPTM1L), 6p21.33 and 15q25.1 (CHRNA5-CHRNA3) variation and lung cancer risk in never-smokers. Carcinogenesis. 2010 Feb;31(2):234-8. doi: 10.1093/carcin/bgp287. Epub 2009 Dec 2. PMID: 19955392.
15. Niu X, Chen Z, Shen S, Liu Y, Zhou D, Zhang J, Li Z, Yu Y, Liao M, Lu S, He L. Association of the CHRNA3 locus with lung cancer risk and prognosis in Chinese Han population. J Thorac Oncol. 2010 May;5(5):658-66. doi: 10.1097/JTO.0b013e3181d5e447. PMID: 20234319.

16. Zienolddiny S, Skaug V, Landvik NE, Ryberg D, Phillips DH, Houlston R, Haugen A. The TERT-CLPTM1L lung cancer susceptibility variant associates with higher DNA adduct formation in the lung. Carcinogenesis. 2009 Aug;30(8):1368-71. doi: 10.1093/carcin/bgp131. Epub 2009 May 22. PMID: 19465454.
17. Schwartz AG, Cote ML, Wenzlaff AS, Land S, Amos CI. Racial differences in the association between SNPs on 15q25.1, smoking behavior, and risk of non-small cell lung cancer. J Thorac Oncol. 2009 Oct;4(10):1195-201. doi: 10.1097/JTO.0b013e3181b244ef. PMID: 19641473; PMCID: PMC3768000.
18. Falvella FS, Galvan A, Frullanti E, Spinola M, Calabrò E, Carbone A, Incarbone M, Santambrogio L, Pastorino U, Dragani TA. Transcription deregulation at the 15q25 locus in association with lung adenocarcinoma risk. Clin Cancer Res. 2009 Mar 1;15(5):1837-42. doi: 10.1158/1078-0432.CCR-08-2107. Epub 2009 Feb 17. PMID: 19223495.
19. Amos CI, Wu X, Broderick P, Gorlov IP, Gu J, Eisen T, Dong Q, Zhang Q, Gu X, Vijayakrishnan J, Sullivan K, Matakidou A, Wang Y, Mills G, Doheny K, Tsai YY, Chen WV, Shete S, Spitz MR, Houlston RS. Genome-wide association scan of tag SNPs identifies a susceptibility locus for lung cancer at 15q25.1. Nat Genet. 2008 May;40(5):616-22. doi: 10.1038/ng.109. Epub 2008 Apr 2. PMID: 18385676; PMCID: PMC2713680.
20. Broderick P, Wang Y, Vijayakrishnan J, Matakidou A, Spitz MR, Eisen T, Amos CI, Houlston RS. Deciphering the impact of common genetic variation on lung cancer risk: a genome-wide association study. Cancer Res. 2009 Aug 15;69(16):6633-41. doi: 10.1158/0008-5472.CAN-09-0680. Epub 2009 Aug 4. PMID: 19654303; PMCID: PMC2754318.
21. Jaworowska E, Trubicka J, Lener MR, Masojć B, Złowocka-Perłowska E, McKay JD, Renard H, Oszutowska D, Wokołorczyk D, Lubiński J, Grodzki T, Serwatowski P, Nej-Wołosiak K, Tołoczko-Grabarek A, Sikorski A, Słojewski M, Jakubowska A, Cybulski C, Lubiński J, Scott RJ. Smoking related cancers and loci at chromosomes 15q25, 5p15, 6p22.1 and 6p21.33 in the Polish population. PLoS One. 2011;6(9):e25057. doi: 10.1371/journal.pone.0025057. Epub 2011 Sep 22. PMID: 21966413; PMCID: PMC3178595.
22. Ji X, Gui J, Han Y, Brennan P, Li Y, McKay J, Caporaso NE, Bertazzi PA, Landi MT, Amos CI. The role of haplotype in 15q25.1 locus in lung cancer risk: results of scanning chromosome 15. Carcinogenesis. 2015 Nov;36(11):1275-83. doi: 10.1093/carcin/bgv118. Epub 2015 Aug 16. PMID: 26282330; PMCID: PMC4635666.
23. Kohno T, Kunitoh H, Mimaki S, Shiraishi K, Kuchiba A, Yamamoto S, Yokota J. Contribution of the TP53, OGG1, CHRNA3, and HLA-DQA1 genes to the risk for lung squamous cell carcinoma. J Thorac Oncol. 2011 Apr;6(4):813-7. doi: 10.1097/JTO.0b013e3181ee80ef. PMID: 21623257.
24. Lips EH, Gaborieau V, McKay JD, Chabrier A, Hung RJ, Boffetta P, Hashibe M, Zaridze D, Szeszenia-Dabrowska N, Lissowska J, Rudnai P, Fabianova E, Mates D, Bencko V, Foretova L, Janout V, Field JK, Liloglou T, Xinarianos G, McLaughlin J, Liu G, Skorpen F, Elvestad MB, Hveem K, Vatten L, Study E, Benhamou S, Lagiou P, Holcátová I, Merletti F, Kjaerheim K, Agudo A, Castellsagué X, Macfarlane TV, Barzan L, Canova C, Lowry R, Conway DI, Znaor A, Healy C, Curado MP, Koifman S, Eluf-Neto J, Matos E, Menezes A, Fernandez L, Metspalu A, Heath S, Lathrop M, Brennan P. Association between a 15q25 gene variant, smoking quantity and tobacco-related cancers among 17 000 individuals. Int J Epidemiol. 2010 Apr;39(2):563-77. doi: 10.1093/ije/dyp288. Epub 2009 Sep 23. PMID: 19776245; PMCID: PMC2913450.
25. Shiraishi K, Kohno T, Kunitoh H, Watanabe S, Goto K, Nishiwaki Y, Shimada Y, Hirose H, Saito I, Kuchiba A, Yamamoto S, Yokota J. Contribution of nicotine acetylcholine receptor polymorphisms to lung cancer risk in a smoking-independent manner in the Japanese. Carcinogenesis. 2009 Jan;30(1):65-70. doi: 10.1093/carcin/bgn257. Epub 2008 Nov 12. PMID: 19005185.
26. Tekpli X, Landvik NE, Skaug V, Gulsvik A, Haugen A, Zienolddiny S. Functional effect of polymorphisms in 15q25 locus on CHRNA5 mRNA, bulky DNA adducts and TP53 mutations. Int J Cancer. 2013 Apr 15;132(8):1811-20. doi: 10.1002/ijc.27870. Epub 2012 Oct 20. Erratum in: Int J Cancer. 2014 Nov 15;135(10):E12. PMID: 23011884.
27. Truong T, Hung RJ, Amos CI, Wu X, Bickeböller H, Rosenberger A, Sauter W, Illig T, Wichmann HE, Risch A, Dienemann H, Kaaks R, Yang P, Jiang R, Wiencke JK, Wrensch M, Hansen H, Kelsey KT, Matsuo K, Tajima K, Schwartz AG, Wenzlaff A, Seow A, Ying C, Staratschek-Jox A, Nürnberg P, Stoelben E, Wolf J, Lazarus P, Muscat JE, Gallagher CJ, Zienolddiny S, Haugen A, van der Heijden HF, Kiemeney LA, Isla D, Mayordomo JI, Rafnar T, Stefansson K, Zhang ZF, Chang SC, Kim JH, Hong YC, Duell EJ, Andrew AS, Lejbkowicz F, Rennert G, Müller H, Brenner H, Le Marchand L, Benhamou S, Bouchardy C, Teare MD, Xue X, McLaughlin J, Liu G, McKay JD, Brennan P, Spitz MR. Replication of lung cancer susceptibility loci at chromosomes 15q25, 5p15, and 6p21: a pooled analysis from the International Lung Cancer Consortium. J Natl Cancer Inst. 2010 Jul 7;102(13):959-71. doi: 10.1093/jnci/djq178. Epub 2010 Jun 14. PMID: 20548021; PMCID: PMC2897877.
28. Young RP, Hopkins RJ, Hay BA, Epton MJ, Black PN, Gamble GD. Lung cancer gene associated with COPD: triple whammy or possible confounding effect? Eur Respir J. 2008 Nov;32(5):1158-64. doi:

10.1183/09031936.00093908. PMID: 18978134.

29. Lee JY, Yoo SS, Kang HG, Jin G, Bae EY, Choi YY, Choi JE, Jeon HS, Lee J, Lee SY, Cha SI, Kim CH, Park JY. A functional polymorphism in the CHRNA3 gene and risk of chronic obstructive pulmonary disease in a Korean population. J Korean Med Sci. 2012 Dec;27(12):1536-40. doi: 10.3346/jkms.2012.27.12.1536. Epub 2012 Dec 7. PMID: 23255854; PMCID: PMC3524434.

Supplementary Table S4. Associations between variants in the *CHRNA* genes with cancer or noncancerous diseases risk.

ID	Disease	Gene	Variant	Alleles[a]	Ethinicity	Number evaluate		Genetic models	Effect model	Risk of Meta-analysis		Heterogeneity		Venice Criteria[b]	Venice Criteria Grade	Amount of evidence		Replication	Protection from bias		First study	Reason for bias exemption	Deviation from HWE	Pegger	Pbegger	Low OR	FPRP values[c]	Credibility of Evidence[d]
						Datasets	Cases/Controls			OR (95%CI)	*P* value	I^2(%)	P_Q			N_{minor}	Grade		Grade	Reasons for bias								
1	COPD	CHRNA3	rs1051730	A vs G	All	4	1618/1909	Allelic	Fixed	1.630 (1.293-2.054)	< 0.001	0.0	0.897	BAA	Moderate	323	B	A	A	No	No	Na	No	0.740	0.308	No	<0.001	Strong
								Dominant	Fixed	1.662 (1.300-2.124)	< 0.001	0.0	0.877	BAA	Moderate	312	B	A	A	No	No	Na	No	0.904	0.308	No	<0.001	Strong
								Recessive	Fixed	2.262 (0.740-6.916)	0.152	0.0	0.425															
					Asian	3	1501/1717	Allelic	Fixed	1.591 (1.204-2.103)	0.001	0.0	0.780	BAA	Moderate	212	B	A	A	No	No	Na	No	0.927	1.000	No	0.001	Strong
								Dominant	Fixed	1.625 (1.222-2.160)	0.001	0.0	0.748	BAA	Moderate	210	B	A	A	No	No	Na	No	0.975	1.000	No	<0.001	Strong
								Recessive	Fixed	1.017 (0.143-9.229)	0.987	0.0	0.341															
2	COPD	CHRNA3	rs6495309	T vs C	Asian	3	1917/2068	Allelic	Fixed	0.830 (0.759-0.906)	< 0.001	25.9	0.259	ABA	Moderate	3632	A	B	A	No	No	Na	No	0.543	1.000	No	<0.001	Strong
								Dominant	Fixed	0.736 (0.644-0.842)	< 0.001	0.0	0.892	AAA	Strong	2725	A	A	A	No	No	Na	No	0.593	1.000	No	<0.001	Strong
								Recessive	Random	0.830 (0.637-1.081)	0.166	64.7	0.059															
3	COPD	CHRNA5	rs16969968	A vs G	All	6	3126/7685	Allelic	Fixed	1.307 (1.205-1.417)	< 0.001	0.0	0.471	AAC	Weak	5236	A	A	C	publication bias	No	Na	No	0.007	0.452	No	<0.001	Moderate
								Dominant	Fixed	1.413 (1.268-1.573)	< 0.001	0.0	0.687	AAC	Weak	4375	A	A	C	publication bias	No	Na	No	0.025	0.707	No	<0.001	Moderate
								Recessive	Fixed	1.370 (1.154-1.625)	< 0.001	0.0	0.713	BAA	Moderate	861	B	A	A	No	No	Na	No	0.637	0.806	No	<0.001	Strong
					Asian	3	1501/1717	Allelic	Fixed	1.591 (1.204-2.103)	0.001	0.0	0.780	BAA	Moderate	212	B	A	A	No	No	Na	No	0.927	1.000	No	0.001	Strong
								Dominant	Fixed	1.625 (1.222-2.160)	0.001	0.0	0.748	BAA	Moderate	210	B	A	A	No	No	Na	No	0.975	1.000	No	<0.001	Strong
								Recessive	Fixed	1.017 (0.143-7.229)	0.987	0.0	0.341															
4	Lung cancer	CHRNA3	rs1051730	A vs G	All	11	7657/7515	Allelic	Fixed	1.348 (1.276-1.424)	< 0.001	37.0	0.103	ABA	Moderate	8453	A	B	A	publication bias	No	GWAS	No	0.015	0.087	No	<0.001	Strong
								Dominant	Fixed	1.446 (1.342-1.559)	< 0.001	10.0	0.349	AAA	Strong	6939	A	A	A	publication bias	No	GWAS	No	0.006	0.020	No	<0.001	Strong
								Recessive	Fixed	1.519 (1.356-1.700)	< 0.001	0.0	0.582	AAA	Strong	1514	A	A	A	No	No	GWAS	No	0.546	0.917	No	<0.001	Strong
					Asian	3	1834/1460	Allelic	Fixed	2.280 (1.626-3.197)	< 0.001	0.0	0.978	BAA	Moderate	177	B	A	A	No	No	GWAS	No	0.103	0.296	No	<0.001	Strong
								Dominant	Fixed	2.329 (1.649-3.291)	< 0.001	0.0	0.982	BAA	Moderate	173	B	A	A	publication bias	No	GWAS	No	0.037	0.296	No	<0.001	Strong
								Recessive	Random	2.249 (0.234-21.659)	0.483	Na	Na															
					Caucasian	6	3492/5434	Allelic	Fixed	1.313 (1.240-1.390)	< 0.001	0.0	0.611	AAA	Strong	7847	A	A	A	No	No	GWAS	No	0.399	0.452	No	<0.001	Strong
								Dominant	Fixed	1.389 (1.283-1.504)	< 0.001	0.0	0.886	AAA	Strong	6428	A	A	A	No	No	GWAS	No	0.944	1.000	No	<0.001	Strong
								Recessive	Fixed	1.506 (1.344-1.688)	< 0.001	8.0	0.365	AAA	Strong	1419	A	A	A	No	No	GWAS	No	0.216	0.133	No	<0.001	Strong
5	Lung cancer	CHRNA3	rs12914385	T vs C	All	4	10037/4443	Allelic	Random	1.096 (0.799-1.505)	0.569	96.7	< 0.001															
								Dominant	Random	1.101 (0.712-1.703)	0.665	96.6	< 0.001															
								Recessive	Random	1.206 (0.839-1.733)	0.312	89.4	< 0.001															
					Caucasian	3	8514/2900	Allelic	Random	1.264 (1.053-1.517)	0.012	84.4	0.002	ACA	Weak	9678	A	C	A	No	No	Na	No	0.258	1.000	No	0.012	Moderate
								Dominant	Random	1.458 (1.331-1.596)	0.014	81.5	0.004	ACA	Weak	7594	A	C	A	No	No	Na	No	0.268	1.000	No	<0.001	Moderate
								Recessive	Random	1.449 (1.134-1.851)	0.003	66.5	0.051	ACA	Weak	2084	A	C	A	No	No	Na	No	0.234	1.000	No	0.003	Moderate
6	Lung cancer	CHRNA3	rs3743073	T vs G	All	3	1391/1500	Allelic	Random	0.785 (0.546-1.129)	0.191	91.3	< 0.001															
								Dominant	Random	0.755 (0.410-1.392)	0.368	90.7	< 0.001															
								Recessive	Random	0.729 (0.504-1.056)	0.094	80.0	0.007															
7	Lung cancer	CHRNA3	rs578776	A vsG	All	3	1254/2009	Allelic	Fixed	0.868 (0.773-0.976)	0.018	0.0	0.908	AAA	Strong	2089	A	A	A	No	No	Na	No	0.982	1.000	No	0.018	Strong
								Dominant	Fixed	0.841 (0.722-0.979)	0.026	0.0	0.937	AAA	Strong	1667	A	A	A	No	No	Na	No	0.950	1.000	No	0.025	Strong
								Recessive	Random	0.838 (0.655-1.073)	0.161	0.0	0.944															
8	Lung cancer	CHRNA3	rs6495309	T vs C	Asian	3	1865/1983	Allelic	Fixed	0.770 (0.704-0.843)	< 0.001	0.0	0.400	AAA	Strong	3427	A	A	A	No	No	Na	No	0.627	1.000	No	<0.001	Strong
								Dominant	Fixed	0.736 (0.642-0.843)	< 0.001	0.0	0.749	AAA	Strong	2633	A	A	A	No	No	Na	No	0.669	1.000	No	<0.001	Strong
								Recessive	Fixed	0.680 (0.580-0.797)	< 0.001	0.0	0.373	BAA	Moderate	794	B	A	A	No	No	Na	No	0.640	1.000	No	<0.001	Strong
9	Lung cancer	CHRNA3	rs8042374	G vs A	Caucasian	3	8501/2920	Allelic	Random	0.814 (0.703-0.941)	0.006	67.7	0.045	ACC	Weak	4610	A	C	C	publication bias	No	Na	No	0.067	0.296	No	0.005	Moderate
								Dominant	Fixed	0.763 (0.697-0.834)	< 0.001	47.3	0.150	ABC	Weak	4124	A	B	C	publication bias	No	Na	No	0.015	0.296	No	<0.001	Moderate
								Recessive	Random	0.715 (0.503-1.016)	0.061	62.6	0.069															
10	Lung cancer	CHRNA3	rs938682	A vs G	All	3	4958/2575	Allelic	Fixed	1.239 (1.131-1.357)	< 0.001	0.0	0.384	AAC	Weak	11851	A	A	C	publication bias	No	Na	No	0.037	0.296	No	<0.001	Moderate
								Dominant	Random	1.233 (0.829-1.834)	0.301	61.8	0.073															
								Recessive	Fixed	1.295 (1.158-1.447)	< 0.001	0.0	0.932	AAA	Strong	4676	A	A	A	No	No	Na	No	0.204	1.000	No	<0.001	Strong
11	Lung cancer	CHRNA5	rs16969968	A vs G	All	14	22794/82907	Allelic	Fixed	1.293 (1.260-1.328)	< 0.001	25.1	0.183	ABA	Moderate	72181	A	B	A	No	No	Na	No	0.597	0.743	No	<0.001	Strong
								Dominant	Fixed	1.374 (1.324-1.426)	< 0.001	34.4	0.100	ABA	Moderate	59246	A	B	A	No	No	Na	No	0.720	1.000	No	<0.001	Strong
								Recessive	Fixed	1.445 (1.372-1.522)	< 0.001	0.0	0.568	AAA	Strong	12935	A	A	A	No	No	Na	No	0.417	0.584	No	<0.001	Strong
					Caucasian	12	21035/80599	Allelic	Fixed	1.298 (1.264-1.333)	< 0.001	0.0	0.575	AAC	Weak	71859	A	A	C	publication bias	No	Na	No	0.088	1.000	No	<0.001	Moderate
								Dominant	Fixed	1.384 (1.333-1.437)	< 0.001	0.0	0.495	AAA	Strong	58937	A	A	A	No	No	Na	No	0.202	0.732	No	<0.001	Strong
								Recessive	Fixed	1.447 (1.374-1.524)	< 0.001	0.0	0.540	AAC	Weak	12922	A	A	C	publication bias	No	Na	No	0.091	0.837	No	<0.001	Moderate
12	Lung cancer	CHRNA5	rs588765	C vs T	Caucasian	4	5851/7321	Allelic	Fixed	1.124 (1.069-1.182)	< 0.001	0.0	0.890	AAC	Weak	15882	A	A	C	publication bias,	No	Na	No	0.062	0.308	YES	<0.001	Moderate
								Dominant	Fixed	1.122 (1.020-1.234)	0.018	0.0	0.724	AAC	Weak	11085	A	A	C	first study, Low OR	YES	Na	No	0.267	0.308	YES	0.018	Moderate
								Recessive	Fixed	1.192 (1.109-1.280)	< 0.001	0.0	0.898	AAA	Strong	4797	A	A	A	No	No	Na	No	0.574	0.734	No	<0.001	Strong

Abbreviations:OR, odds ratio; A, adenine; T, thymine; G, guanine; C, cytosine; COPD: chronic obstructive pulmonary disease

First study=Yes: Significance lost excluding the first published or first positive report; No: Significance was not lost excluding the first published or first positive report;

HWE:=Yes: Significance lost excluding studies with controls violating HWE; No: A meta-analysis didn't include studies with controls violating HWE.

Low OR=Low magnitude of the association (i.e. 0.87<OR<1.15)

Publication bias= Possible existence of potential publication bias (Begg's test, p<0.10)

Small study (Sample size<100) = Possible existence of potential small study effect (modified Egger's test, p<0.10).

[a]Minor alleles vs major alleles (reference).

[b]Strength of epidemiological evidence based on the Venice criteria.

[c]The prior probability of FPRP is 0.05, and the FPRP level of noteworthiness is 0.20.

[d]Degree of epidemiological credibility based on the combination of results from Venice guidelines and FPRP tests.

Supplementary Table S5. Analyses of expression quantitative trait locus (eQTL) in significant variants associated with risk of disease.

Variant	Gene	Tissue	*P* value	Effect Size
rs1051730	CHRNA5	Lung	5.6×10^{-9}	-0.33
	CHRNA3	Lung	4.5×10^{-05}	-0.25
rs6495309	IREB2	Lung	1.2×10^{-05}	0.094
rs578776	IREB2	Lung	5.0×10^{-05}	0.082
rs938682	IREB2	Lung	4.4×10^{-06}	-0.096
rs16969968	CHRNA5	Lung	5.0×10^{-09}	-0.33
	CHRNA3	Lung	1.0×10^{-04}	-0.25
rs588765	CHRNA5	Lung	1.4×10^{-12}	-0.38
	CHRNA3	Lung	5.0×10^{-05}	-0.24

Data source: Genotype-Tissue Expression (GTEx) Project

Supplementary Table S6. Linkage Disequilibrium (r^2) among the significant variants in the *CHRNA* genes

SNP Pair[1]	Asian[2]	European[2]	African[2]
rs1051730 & rs6495309	0.0166	0.1751	0.0298
rs1051730 & rs578776	0.1105	0.2200	0.0064
rs1051730 & rs938682	0.0241	0.1871	0.0450
rs6495309 & rs578776	0.1698	0.7566	0.1289
rs6495309 & rs938682	0.8543	0.9361	0.5143
rs578776 & rs938682	0.2185	0.8088	0.2991
rs16969968 & rs588765	0.0057	0.3601	0.0080
rs16969968 & rs1051730	1.0000	0.9872	0.2520
rs16969968 & rs6495309	0.0166	0.1729	0.0086
rs16969968 & rs578776	0.1105	0.2258	0.0366
rs16969968 & rs938682	0.0241	0.1847	0.0113
rs588765 & rs1051730	0.0057	0.3648	0.0316
rs588765 & rs6495309	0.1170	0.1856	0.0859
rs588765 & rs578776	0.6089	0.1089	0.4502
rs588765 & rs938682	0.1516	0.1985	0.1477

[1]Data source: ldlink.nci.nih.gov
[2]Asians, European, and African were selected from five major population categories provided by the databases.
